# A positive feedback loop between BACH1 and IL-1β promotes the progression of HPV-negative head and neck squamous cell carcinoma

**DOI:** 10.1186/s12964-026-02957-2

**Published:** 2026-05-25

**Authors:** Zishanbai Zhang, Miao Wang, Wenjuan Wang, Minghui Zhao, Yiming Ding, Tingyao Ma, Zhaohan Zhang, Xilin Wang, Yuchen Xiang, Yaning Wang, Wenying Zhong, Zicheng Wu, Yiran Meng, Xiaohong Chen, Lin Shan

**Affiliations:** 1 Department of Otolaryngology Head and Neck Surgery, Beijing Tongren Hospital, Capital Medical University, Key Laboratory of Otolaryngology Head and Neck Surgery, Ministry of Education, Capital Medical University, Dong Jiao Min Xiang Street, Beijing, China; 2 Department of Biochemistry and Molecular Biology, School of Basic Medical Sciences, Laboratory for Clinical Medicine, Capital Medical University, 10 You’anmen Outer West 1 st Street, Beijing, 100069 China; 3 Department of Otorhinolaryngology Head and Neck Surgery, Beijing Children’s Hospital, Capital Medical University, National Center for Children’s Health, Beijing, China

## Abstract

Human papillomavirus (HPV)-negative head and neck squamous cell carcinoma (HNSCC) is associated with a poorer prognosis compared to its HPV-positive counterpart; however, the underlying regulatory molecular mechanisms driving its progression remain poorly understood. In this study, we identify BTB Domain and CNC Homolog 1 (BACH1) as a critical oncogenic regulator that is upregulated in HPV-negative HNSCC. We demonstrate that BACH1 promotes tumor progression by enhancing the proliferation and inhibiting apoptosis of cancer cells. Mechanistically, BACH1 is associated with the SWI/SNF chromatin remodeling complex and transcriptionally activates downstream target genes, including *IL1B*, thereby inducing a transcriptional reprogramming that promotes proliferation and resists apoptosis. Notably, BACH1-driven upregulation of interleukin-1β (IL-1β) establishes a positive feedback loop that sustains oncogenic signaling. Pharmacological disruption of this axis using the IL-1 receptor antagonist Anakinra significantly attenuates tumor growth in vitro* and *in vivo. Clinically, co-upregulation of BACH1, BRG1, and IL-1β is correlated with reduced overall survival in patients with HPV-negative HNSCC. Collectively, our findings characterize the BACH1–IL-1β signaling axis as a prognostic biomarker and highlight IL-1R blockade as a promising therapeutic strategy for the treatment of HPV-negative HNSCC.

**Supplementary Information:**

The online version contains supplementary material available at 10.1186/s12964-026-02957-2.

## Introduction

Head and neck squamous cell carcinoma (HNSCC) ranks as the sixth most common malignant tumor worldwide, with over 890,000 new cases diagnosed annually. The global incidence of HNSCC is projected to rise further, underscoring the increasing burden of this disease [1]. HNSCC is characterized by pronounced molecular heterogeneity and is clinically stratified into two etiologically distinct subtypes: human papillomavirus (HPV)-positive and HPV-negative tumors. Notably, HPV-negative HNSCC is associated with significantly poorer clinical outcomes, including higher incidence and recurrence rate, diminished responsiveness to radiotherapy, chemotherapy and neoadjuvant therapy, and a markedly lower 5-year overall survival rate and 5-year disease-free survival rate [2–4]. To date, effective targeted therapies for HPV-negative HNSCC remain unavailable [5]. Given these clinical challenges, a deeper understanding of the molecular regulatory mechanisms underlying HPV-negative HNSCC, along with identification of novel therapeutic targets, is crucial for improving patient prognosis and expanding treatment options for this devastating disease.

BACH1 (BTB and CNC homolog 1), a member of the CNC-bZIP transcription factor family, exhibits oncogenic properties through aberrant overexpression in various malignancies, including esophageal squamous cell carcinoma [6], pancreatic cancer [7], hepatocellular carcinoma [8] and non-small cell lung cancer [9]. Elevated BACH1 expression is closely associated with poor prognosis across these cancers [6, 10, 11]. Mechanistically, BACH1 overexpression disrupts several key cellular processes, including heme homeostasis, cell cycle regulation, and oxidative stress responses, to result in metabolic reprogramming and epithelial-mesenchymal transition (EMT), thereby promoting tumor cell proliferation, invasion, and metastasis [7, 12–14]. In addition to its cell-intrinsic oncogenic roles, BACH1 plays a pivotal role in modulating the tumor microenvironment (TME) [14]. In glioblastoma, high BACH1 expression is positively correlated with increased secretion of tumor-associated macrophage (TAM)-recruiting chemokines such as MCP1, mGM-CSF, and EGF. Furthermore, it is correlated with elevation of markers associated with M2-polarized TAMs and the upregulation of multiple immune checkpoints (ICs), including CD276, TIM-3, LAG3, TIGIT, and LGALS9, collectively contributing to the establishment of an immunosuppressive TME [15]. Despite the well-documented oncogenic role of BACH1 in several cancer types, its biological function and underlying molecular mechanisms in HNSCC remain underexplored. Notably, under physiological conditions, Hemin facilitates the degradation of BACH and is considered a natural negative regulator of BACH1 activity [16], underscoring the importance of elucidating the role of BACH1 in the progression of HNSCC and evaluating the potential of Hemin as a therapeutic agent for this malignancy.

The SWI/SNF complex, an ATP-dependent chromatin remodeling complex, plays a pivotal role in regulating gene expression, tumorigenesis and progression. Mutations in one or more SWI/SNF subunits are present in approximately 25% of all human cancers [17]. Among these subunits, BRG1 (also known as SMARCA4) functions as a key catalytic component. BRG1 is frequently upregulated in several malignancies, including breast, colorectal, and prostate cancers, and its overexpression is associated with poor patient prognosis [18, 19]. Additionally, BRG1 deficiency has been shown to impairs autophagy and inhibit apoptosis, thereby promoting tumor progression [20, 21]. In triple-negative breast cancer, BRG1 facilitates EMT and contributes to cisplatin resistance through activation of the YAP pathway [22]. The complex manifests as three major variants: canonical BAF (cBAF), non-canonical BAF (ncBAF), and polybromo associated BAF (PBAF). Despite sharing the essential ATPase module, each variant possesses distinct subunits that dictate its unique functional profile and genomic targeting, such as the preference of PBAF for promoters and cBAF for enhancers [23] [24]. Nevertheless, despite these insights, the specific roles and molecular mechanisms of these SWI/SNF subtypes in HPV-negative HNSCC progression remain inadequately understood.

Interleukin-1β(IL-1β), a pleiotropic proinflammatory cytokine, contributes to tumor progression through multiple mechanisms [25, 26]. Early studies primarily focus on its role in driving metabolic reprogramming in immune cells in response to chronic inflammatory stimulation. IL-1β is predominantly secreted by activated monocytes and macrophages [27]. Upon pathogen or inflammatory stimulation, IL-1β production is initiated through the activation of pattern recognition receptors such as Toll-like receptors (TLRs), which subsequently activate inflammasomes, like NLRP3. This leads to caspase-1-mediated cleavage of the pro-form of IL-1β into its mature, bioactive form, which is then secreted into the extracellular environment [28]. Once released, IL-1β promotes the recruitment of immunosuppressive cell populations, including regulatory T cells (Tregs) and myeloid-derived suppressor cells (MDSCs), facilitating the development of an immunosuppressive tumor microenvironment that promotes immune evasion and tumor progression [29, 30]. In recent years, increasing evidence suggests that IL-1β can also be secreted by tumor cells themselves, where it plays a pivotal role in supporting tumor cell survival, proliferation, and metastasis. These effects are mediated through activation of key oncogenic signaling pathways, including the MAPK and PI3K/AKT cascades [31, 32]. Despite these advances, the biological functions and molecular mechanisms of IL-1β in HNSCC remain largely undefined and warrant further investigation.

In this study, we identified that BACH1 expression is significantly elevated in HPV-negative HNSCC compared to HPV-positive HNSCC and is strongly associated with poor prognosis of patients. Mechanistically, we demonstrated that BACH1 recruits the SWI/SNF complex to transcriptionally activate genes such as *IL1B*. This activation subsequently induces transcriptional reprogramming of downstream targets including *MDM2*, *SIRT1*, and *USP13*, thereby promoting proliferation and suppressing apoptosis of tumor cells. Notably, IL-1β also enhances BACH1 expression, establishing a positive feedback loop that further accelerates the progression of HPV-negative HNSCC. Importantly, we demonstrated that Hemin, a known inhibitor of BACH1, is insufficient to suppress tumor progression, but the IL-1β receptor antagonist Anakinra effectively inhibits proliferation and induces apoptosis of tumor cells, highlighting its potential as a therapeutic strategy to disrupt feedback loop. We explored the clinicopathological significance of BACH1-IL-1β axis in HPV-negative HNSCC.

## Materials and methods

### Antibodies and reagents

The antibodies used in this study were as follows: anti-BACH1 (14018–1-AP, for western blotting [WB], immunoprecipitation [IP], immunohistochemistry [IHC], quantitative chromatin immunoprecipitation [qChIP], and cleavage under targets and tagmentation [CUT&Tag]), anti-Alpha Tubulin (66031–1-lg, for WB), anti-IL-1β (16806–1-AP, for WB), anti-PBRM1 (12563–1-AP, for WB and IP), anti-SMARCA5/SNF2H (13066–1-AP for WB), anti-SIRT1 (13161–1-AP for WB), anti-USP13 (16840–1-AP for WB), anti-MDM2 (27883–1-AP for WB), anti-p38 MAPK (14064–1-AP for WB), anti-phospho-p38 MAPK (Thr180/Tyr182) (28796–1-AP for WB and IHC), anti-AKT (10176–2-AP for WB), and anti-phospho-AKT (Ser473) (66444–1-Ig for WB and IHC) from Proteintech; anti-phospho-NF- kB p65 (Ser536) (F0155 for WB and IHC) and anti-NF-κB p65 (F0006 for WB) from Selleck; anti-BRG1 (ab110641, for WB, IP, qChIP and CUT&Tag), anti-ARID1A (ab182560, for WB and IP), and anti-SMARCA5/SNF2H (A2000 for IP) from Abclone; anti-Cleaved- IL-1β (83186 T for WB) from Cell Signaling Technology. The 3 × FLAG peptide (F4799) was purchased from Macgene. *InVivo*MAb anti-mouse/rat IL-1β (BE0246) was purchased from Bioxcell.

### Plasmids

FLAG-tagged BACH1 and the ΔBTB BACH1 mutant were expressed using the Tag2B vector, whereas the ΔZIP and ΔCNC BACH1 mutants were expressed using the pcDNA3.1 vector. The corresponding empty vectors were used as controls. All plasmids were purchased from YouBio.

### Cell culture

FaDu, CAL 27 and HEK 293 T cell lines were obtained from the American Type Culture Collection (ATCC). FaDu cells were cultured in MEM, CAL 27 cells were cultured in DMEM, and HEK 293 T cells were cultured in 1640 medium, each supplemented with 10% fetal bovine serum (FBS), 100 U/mL penicillin, and 100 U/mL streptomycin (Gibco).

### EdU incorporation assay

FaDu cells stably infected with lentivirus encoding scramble (SCR) or BACH1, as well as shSCR or shBACH, were seeded into 96-well plates at a density of 10^5^ cells/mL and cultured overnight. Cell proliferation during the logarithmic growth phase was assessed using an EdU assay kit (RiboBio), following the manufacturer’s instructions. All experiments were performed in triplicate.

### Colony formation assay

Cells were stably transfected with the indicated expression constructs or specific shRNA-expressing lentiviruses. After seeding, cells were cultured for 7 to 14 days. Colonies were fixed with 4% paraformaldehyde for 15 min, stained with crystal violet, and counted under a light microscope. All experiment were performed in triplicate.

### Tumor xenografts

FaDu cells stably infected with lentivirus encoding SCR or BACH1, as well as shSCR or shBACH, were harvested, and 5 × 10^6^ viable cells in 100 μl PBS were injected into 6–8 weeks old female athymic nude mice (BALB/c; Charles River, Beijing, China). For Hemin treatment experiments, 5 × 10^6^ FaDu cells were injected into the right dorsal flank of 6–8 weeks old female mice to establish subcutaneous xenograft tumors. Mice were randomly assigned to two groups and administered either vehicle or Hemin daily via intraperitoneal (i.p.) injection for 24 consecutive days. For Anakinra treatment experiments, 5 × 10^6^ FaDu cells were injected into the right dorsal flank of 6–8 weeks old female mice to establish subcutaneous xenograft tumors. After tumors reached 50–100 mm^3^, mice were randomized (*n* = 4–6 per group) and treated with daily intraperitoneal injections of Anakinra (25 mg/kg/day) or vehicle control for 12 days. All measurements were taken by investigators blinded to the treatment groups. Tumor volume was calculated using the formula (length × width^2^)/2.

### Immunopurification and silver staining

HEK 293 T cells were stably transfection with a FLAG-BACH1 expression vector. Cell lysates were prepared using a lysis buffer supplemented with a protease inhibitor cocktail (Roche). Immunoaffinity purification was performed using anti-FLAG M2 affinity gel (Sigma-Aldrich), following the manufacturer’s instructions. Lysates from approximately 5 × 10⁷ cells were applied to a pre-equilibrated FLAG affinity column (1 mL bed volume), allowing the FLAG-tagged protein complex to bind to the resin. After binding, the column was washed with cold PBS containing 0.2% Nonidet P-40 to remove non-specifically bound proteins. FLAG peptide (Sigma-Aldrich) was used to elute the bound protein complexes, as per the manufacturer’s protocol. Eluates were resolved by SDS-PAGE using a NuPAGE 4–12% Bis–Tris gel (Invitrogen), followed by silver staining (Pierce). Distinct protein bands were excised and subjected to liquid chromatography-tandem mass spectrometry (LC–MS/MS) for protein identification.

### Co-immunoprecipitation (Co-IP)

Cells were lysed using NETN buffer (150 mM NaCl, 2 mM EDTA, 50 mM Tris–HCl, pH 8.0, 0.2% Nonidet P-40) at 4 °C for 20 min. The supernatant was collected by centrifugation and used as the protein sample. Approximately 500 μg of protein was incubated with either control or specific antibodies (1–2 μg) at 4 °C for 12 h. Subsequently, 50 μL of 50% protein G magnetic beads were added and incubated for an additional 2 h. beads were washed five times with NETN buffer, then resuspended in 2 × SDS-PAGE sample buffer and boiled at 100 °C for 10 min to elute the bound proteins. Western blotting analysis was performed following SDS-PAGE using the target antibodies.

### Cut&tag

CUT&Tag was performed using the Hyperactive Universal CUT&Tag Assay Kit for Illumina (TD903, Vazyme) following the manufacturer’s protocol. Briefly, 1 × 10^5^ FaDu cells were collected, resuspended in 100 μL wash buffer, and incubated with activated ConA beads for 10 min in an 8-tube strip. A total of 50 μL of pre-chilled antibodies buffer containing antibodies against BACH1, BRG1 or H3K27ac was then added, followed by overnight incubation at 4 °C. After washing, diluted secondary antibodies was added and incubated for 60 min. After three washes with DIG wash buffer, the samples were incubated with diluted pA/G-Tnp transposase for 1 h. Fragmentation was initiated by adding 50 μL diluted TTBL and incubating at 37 °C for 60 min in a thermocycler. DNA was extracted by adding 5 μL proteinase K, 100 μL L/B buffer, and 20 μL DNA extraction beads, followed by a 10-min incubation at 55 °C. After washes, DNA was eluted in sterile ultrapure water and used for library preparation using the TruePrep Index Kit V2 for Illumina (TD202, Vazyme). Libraries were purified with VAHTS DNA Clean Beads (N411, Vazyme), washed with 80% ethanol, and eluted in ultrapure water. Sequencing was performed using the Illumina NovaSeq platform. Data analysis was conducted using the GALAXY platform (https://usegalaxy.org/). Standard quality control metrics—including fragment size distribution and FRiP scores. All data have been uploaded to the GEO database, with the accession number GSE325540.

### ChIP, quantitative ChIP (qChIP), and ChIP/re-ChIP

Eluted DNA was purified using a PCR purification kit (Qiagen). qChIP was performed using the TransStart Top Green qPCR SuperMix (TransGen Biotech) on either an ABI 7500-FAST or LightCycler 480 Real-Time PCR system. Re-ChIP was conducted following the same protocol as the initial IP. Beads from the first IP were eluted with 10 mM DTT at 37 °C for 30 min, diluted 1:50 in dilution buffer (1% Triton X-100, 2 mM EDTA, 150 mM NaCl, 20 mM Tris–HCl; pH 8.1), and subjected to a second IP using the secondary antibodies. The final elution step was performed with 1% sodium dodecyl sulfate in Tris–EDTA (pH 8.0). Primers used for qChIP PCR are listed in Supplementary Table 1.

### RNA Interference

RNA interference was performed using Lipofectamine RNAi MAX (Invitrogen) according to the manufacturer’s protocol. siRNAs were prepared at a final working concentration of 10 nM, and the cells were harvested 72–96 h post-transfection. For each gene, at least three independent siRNA sequences were designed, and the two with the highest knockdown efficiency were used in downstream experiments. siRNA sequences are listed in Supplementary Table 2.

### Quantitative real-time PCR (qPCR)

Total cellular RNA was isolated using the TRIzol reagent (Invitrogen) and subjected to first-strand cDNA synthesis using the reverse transcription system (Roche). All gene transcripts were quantified using the Power SYBR Green PCR Master Mix (Roche) in an ABI PRISM 7500 sequence detection system (Applied Biosystems) or Light Cycler 480 Real-Time PCR system, with *UBC* as the internal control. The list of qPCR primers used is shown in Supplementary Table 3.

### Analysis of transcriptomic data

Head and neck squamous cell carcinoma (HNSCC) transcriptomic data with annotated HPV status were obtained from The Cancer Genome Atlas (TCGA) via the cBioPortal database (http://www.cbioportal.org). Differential gene expression and survival analyses were conducted using the built-in analytical tools available on the platform. Among them, HPV-positive samples were defined based on the presence of E6/E7 gene expression (E6/E7 > 0, *n* = 53), whereas HPV-negative samples were required to have confirmed negative HPV status by both ISH and p16 immunohistochemistry tests (*n* = 56).

### Analysis of single-cell RNA sequencing data

The single-cell RNA sequencing (scRNA-seq) dataset of HNSCC (accession number GSE181919) was downloaded from the Gene Expression Omnibus (GEO, http://www.ncbi.nlm.nih.gov/geo/). Following initial quality control and filtering, the merged dataset was normalized using the NormalizeData function. Highly variable genes were identified with the FindVariableFeatures function. Dimensionality reduction was performed using principal component analysis (PCA), followed by batch effect correction with the Harmony algorithm. Cell clustering was achieved using the FindNeighbors and FindClusters functions, resulting in eight distinct cellular clusters. Marker genes for each cluster were identified using the FindAllMarkers function to facilitate cell type classification. Cluster identities were relabeled using the RenameIdents function based on expression profiles of known marker genes. The HPV status was determined according to the clinical diagnostic information recorded by the original investigators at the time of sample registration. The sample sizes for HPV-positive and HPV-negative patients were 7 and 13, respectively.

### Statistical analysis

The number of independent biological replicates for each experiment. Data are presented as mean ± standard deviation (SD) or mean ± standard error of the mean (SEM), as indicated. Comparisons between two groups were made using two-tailed unpaired Student’s *t*-test, while comparisons among multiple groups were performed using one-way analysis of variance (ANOVA) followed by Bonferroni’s post hoc test. *p*-value of < 0.05 was considered statistically significant. Additionally, data normality was assessed using the Shapiro–Wilk test and the homogeneity of variance was evaluated using Levene’s test to confirm whether the assumptions for parametric tests were met. All statistical analyses were performed using GraphPad Prism 8.0.

### Study approval

All animal handling and experiments were approved by the Animal Care Committee of Capital Medical University. The collection and analysis of human tissue samples were approved by the Medical Ethics Committee of Beijing Tongren Hospital.

### Funding declaration

This study was supported by a grant (82073122 to L.S.) from the National Natural Science Foundation of China, by the Laboratory for Clinical Medicine, Capital Medical University, and by the "Ascending the Peak" Talent Training Program from the Beijing Hospital Management Center (DFL20220201 to X.C.). We thank the Core Facility Center, Capital Medical University, for providing instrumental support.

## Results

### BACH1 is specifically overexpressed in HPV-negative head and neck squamous cell carcinoma

**Fig. 1:**
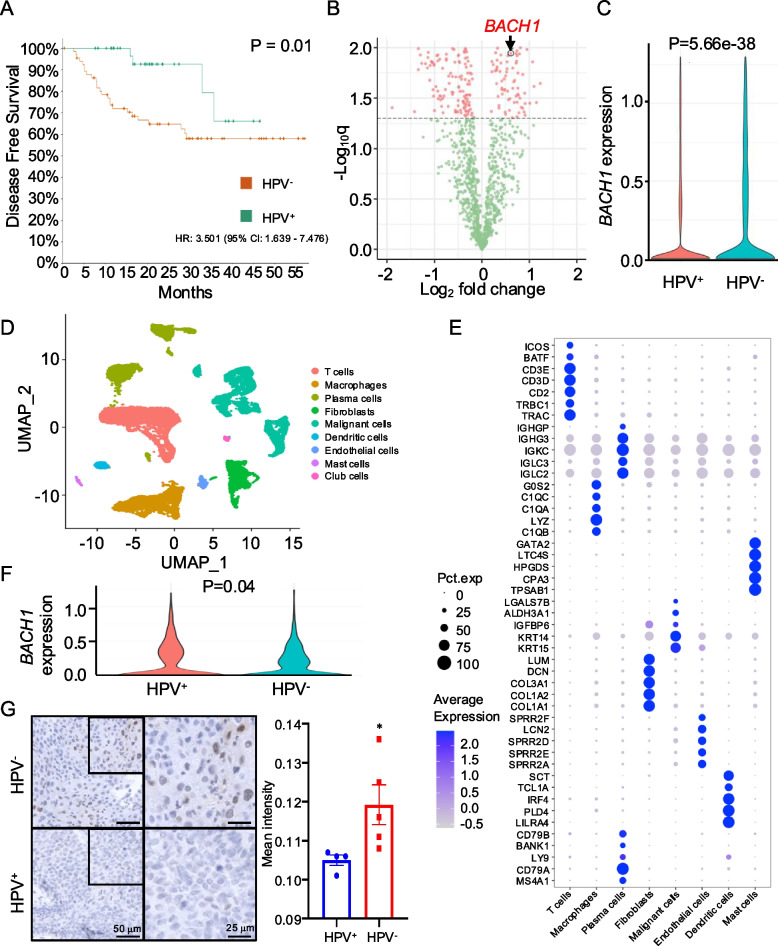
BACH1 is highly expressed in HPV-negative Head and Neck Squamous Cell Carcinoma. **A** Kaplan–Meier survival analysis comparing overall survival between HPV-negative (*n* = 74) and HPV-positive (*n* = 41) head and neck squamous cell carcinoma (HNSCC) patients using the cBioPortal platform (https://www.cbioportal.org). **B** Differential gene expression analysis between HPV-negative (*n* = 74) and HPV-positive (*n* = 41) HNSCC cohorts via cBioPortal. (C) Violin plot showing *BACH1* expression levels in HPV-positive (*n* = 7) versus HPV-negative HNSCC (*n* = 13) samples of the single-cell RNA sequencing (scRNA-seq) dataset GSE181919. (D) Cell clustering identified eight distinct cell populations based on transcriptional profiles in scRNA-seq analysis of dataset GSE181919. (E) Marker gene expression was used to define each of the identified cell populations. (F) Violin plot quantifying *BACH1* expression specifically in malignant cells from both HPV-positive and HPV-negative HNSCC groups. (G) Representative immunohistochemistry (IHC) staining images of BACH1 in formalin-fixed paraffin-embedded (FFPE) tissue samples from HPV-positive (*n* = 4) and HPV-negative (*n* = 5) HNSCC patients. Quantification of average staining intensity was performed using Image-Pro Plus software. Each dot represents a bulk sample from one patient. Error bars represent mean ± SEM (^*^p < 0.05)

To investigate the prognostic relevance of HPV status in head and neck squamous cell carcinoma (HNSCC), we performed Kaplan–Meier survival analysis using TCGA cohort data. The results revealed that the disease-free survival (DFS) of HPV-negative patients was significantly shorter than that of HPV-positive patients (Fig. 1A). To further explore the molecular differences associated with HPV status, we analyzed the expression profiles of transcription factors within the TCGA dataset. Notably, *BACH1* expression was significantly elevated in HPV-negative HNSCC compared to HPV-positive tumors (Fig. 1B), suggesting a potential oncogenic role of *BACH1* in HPV-negative HNSCC. We next analyzed a publicly available single-cell RNA sequencing (scRNA-seq) dataset (GSE181919) comprising 20 HNSCC samples with annotated HPV status (7 HPV-positive and 13 HPV-negative patients). Consistent with the results of the TCGA data analysis, *BACH1* expression was higher in HPV-negative tumors than in HPV-positive ones (Fig. 1C). The corresponding raw expression data are provided in the Supplementary Table 4. Then, cell type annotation based on canonical marker genes identified eight major populations, including T cells, macrophages, plasma cells, fibroblasts, malignant cells, endothelial cells, dendritic cells, and mast cells (Fig. 1D-E). We isolated the malignant cell subclusters for independent analysis. *BACH1* expression remained elevated in HPV-negative malignant cells relative to HPV-positive malignant cells (Fig. 1F). The corresponding raw expression data are provided in the Supplementary Table 5. In order to gain further support of the role of BACH1 in HPV-negative HNSCC progression, we performed immunohistochemical (IHC) staining on clinical specimens from HPV-positive and HPV-negative HNSCC patients. Both IHC staining and quantitation analysis confirmed significantly higher BACH1 expression in HPV-negative samples (Fig. 1G). These results suggest that BACH1 is specifically highly expressed in HPV-negative HNSCC tumors.

**Fig. 2:**
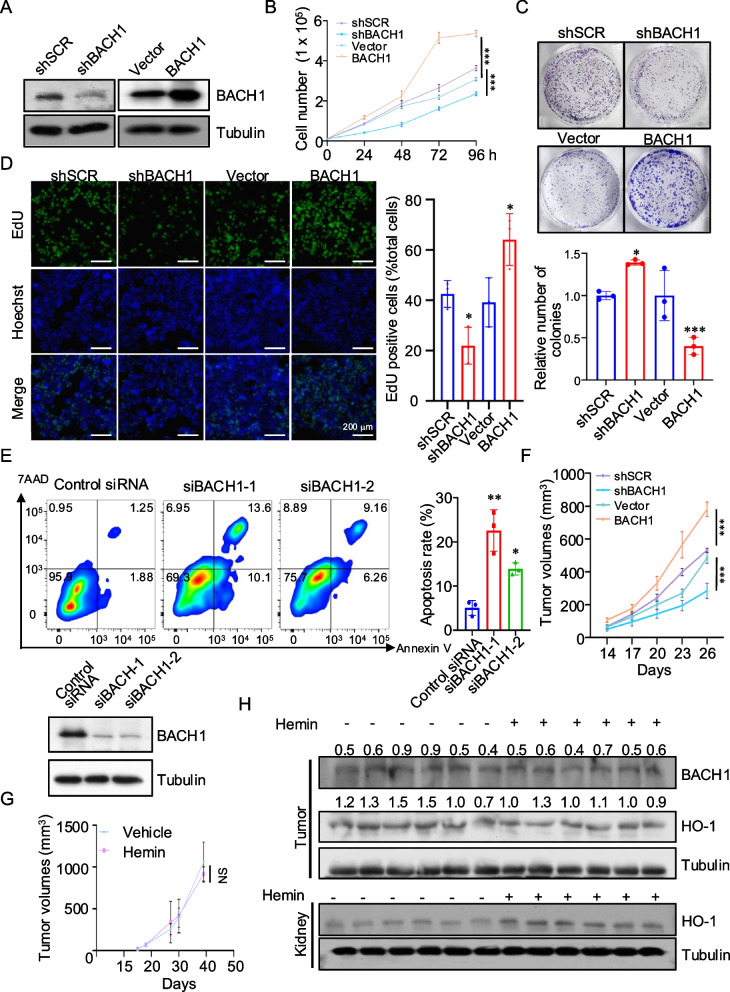
BACH1 promotes proliferation and inhibits apoptosis of HNSCC cells. **A** FaDu cells were infected with lentiviruses carrying the indicated expression constructs for western blotting (WB) with antibodies against BACH1 or Tubulin. **B** Cell proliferation curves of FaDu cells with BACH1 knockdown or overexpression, assessed over time. Error bars, mean ± SD for three independent experiments. (^***^p < 0.001). **C** FaDu cells with BACH1 knockdown or overexpression were cultured for 14 days, followed by crystal violet staining and colony counting. Representative images and quantification are shown. Error bars represent the mean ± SD from three independent experiments. (^*^p < 0.05, ^***^p < 0.001). **D** 5-ethynyl-2’-deoxyuridine (EdU) incorporation assay in FaDu cells with BACH1 knockdown or overexpression. Scale bar: 200 μm. Error bars, mean ± SD for three independent experiments. (^*^p < 0.05). **E** Flow cytometric analysis of apoptosis in FaDu cells with BACH1 knockdown (upper). The knockdown efficiency was validated by WB (lower). Error bars, mean ± SD for three independent experiments. (^**^p < 0.01). **F** Tumor sizes in mice transplanted subcutaneously with BACH1 knockdown or stable overexpression FaDu cells. *n* = 4 animals per group. Error bars represent the mean ± SEM from four independent animals (^***^p < 0.001). **G** Tumor volume in xenograft-bearing female athymic mice treated with either vehicle or Hemin, indicating pharmacological modulation of BACH1 activity in vivo. *n* = 6 animals per group. Error bars represent the mean ± SEM from six independent animals. (H) Following treatment with vehicle or Hemin, kidney and tumor tissues were harvested from mice for WB analysis

To further assess the functional role of BACH1 in HPV-negative HNSCC, we established a stable BACH1 knockdown FaDu cell line (an HPV-negative HNSCC cell line) using lentiviral-delivered short hairpin RNA (shRNA) targeting BACH1. Additionally, a BACH1-overexpressing FaDu cell line was generated via lentiviral transduction with human BACH1 cDNA. Western blotting (WB) analysis confirmed effective knockdown and overexpression of BACH1 (Fig. 2A). Growth curve analysis demonstrated that BACH1 knockdown suppressed FaDu cell proliferation, whereas BACH1 overexpression markedly enhanced cell growth (Fig. 2B). Colony formation assays further demonstrated that BACH1 knockdown significantly reduced colony formation, while BACH1 overexpression increased colony formation (Fig. 2C). Moreover, 5-ethynyl-2’-deoxyuridine (EdU) incorporation assays confirmed that BACH1 knockdown reduced the proliferative capacity of FaDu cells, while BACH overexpression significantly enhanced cell proliferation (Fig. 2D). Apoptosis assessment by Annexin V/7-AAD flow cytometry revealed that BACH1 knockdown markedly increased both early (Annexin V^+^/7-AAD^−^) and late (Annexin V^+^/7-AAD^+^) apoptotic populations (Fig. 2E). To investigate the biological role of BACH1 in promoting the progression of HPV-negative HNSCC in vivo, we subcutaneously injected FaDu cells with stable BACH1 knockdown or overexpression into 6–8 weeks old athymic mice (BABL/c nude; Charles River Laboratories) (*n* = 4). After 2 weeks of monitoring, we observed that BACH1 knockdown significantly reduced primary tumor growth compared to controls, whereas BACH1 overexpression promoted tumor growth (Fig. 2F).

Since Hemin inhibits the biological effects of BACH1 by suppressing its transcriptional activity and protein expression, it is considered a natural degrader of BACH1 [16]. We hypothesized that Hemin could serve as a potential therapeutic agent for HPV-negative HNSCC. To test this hypothesis, we established subcutaneous xenografts by inoculating FaDu cells into 6–8 weeks old athymic mice (n = 6). The mice were randomly divided into two groups and injected intraperitoneally with either vehicle or Hemin every day. Tumor growth was monitored over the next three weeks. Unfortunately, the results indicated that Hemin treatment had minimal effect on the growth of the primary FaDu tumors, suggesting that Hemin is not an effective therapeutic agent for HPV-negative HNSCC (Fig. 2G). Consistent with these findings, WB analysis of tumor tissues revealed no significant reduction in BACH1 protein levels, nor any substantial induction of its target heme oxygenase-1 (HO-1). Quantification of their relative expression is presented in Fig. 2H, indicating that hemin fails to effectively inhibit BACH1 in vivo. Increased expression of HO-1 in the kidney served as a positive control, indicating successful Hemin injection (Fig. 2H). These findings demonstrate that while BACH1 acts as a critical oncogenic driver in HPV-negative HNSCC, pharmacological targeting with its natural inhibitor Hemin does not yield significant antitumor effects in vivo.

### BACH1 is physically associated with the SWI/SNF complex

**Fig. 3:**
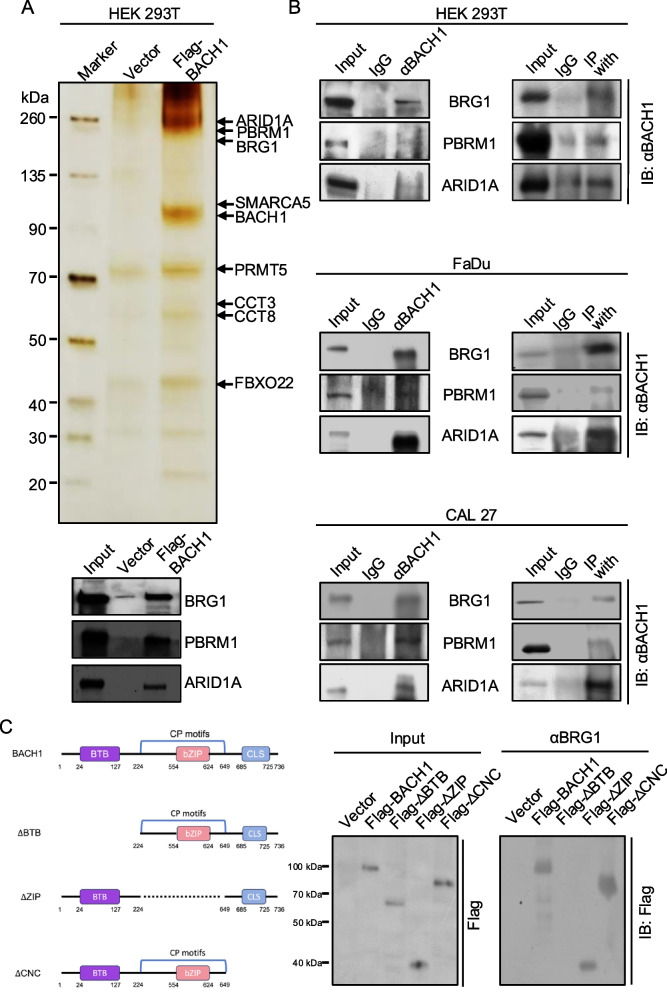
BACH1 is physically associated with the SWI/SNF chromatin remodeling complex. **A** Immunopurification and mass spectrometry analysis of BACH1-associated proteins. Cell extracts were prepared from FLAG-BACH1-expressing HEK 293 T cells and subjected to affinity purification using an anti-FLAG affinity column, followed by elution with FLAG peptide. The eluates were separated by SDS-PAGE and silver stained. Protein bands were excised and analyzed by mass spectrometry (upper). The eluates were analyzed by WB using antibodies against BRG1, PBRM1, SMARCA5, and ARID1A (lower). **B** Whole cell lysates were prepared from HEK 293 T, FaDu, or CAL 27 cells. Immunoprecipitation was performed using antibodies against BACH1, followed by immunoblotting with antibodies against BRG1, PBRM1, and ARID1A. Alternatively, immunoprecipitation was performed with antibodies against the indicated proteins, followed by immunoblotting with antibodies against BACH1. **C** Schematic diagrams of BACH1 and BACH1 deletions are shown (left). FaDu cells were transfected with FLAG-BACH1 or BACH1 deletions. Cellular lysates were immunoprecipitated with anti-BRG1 followed by immunoblotting with anti-FLAG (right)

To comprehensively elucidate the molecular mechanisms underlying BACH1's oncogenic role in tumor progression, we employed an integrated proteomics approach to characterize BACH1-associated protein complexes in vivo. HEK 293 T cells were transfected with FLAG-tagged BACH1 expression plasmid, followed by affinity purification using anti-FLAG M2 magnetic beads and subsequent liquid chromatography-tandem mass spectrometry (LC–MS/MS) analysis. Our mass spectrometry analysis revealed that FLAG-BACH1 co-purifies with several core subunits of the SWI/SNF chromatin remodeling complex. These include the core catalytic ATPase BRG1 (SMARCA4); ARID1A (BAF250A), a component of the canonical BAF (cBAF) complex; and PBRM1 (BAF180), a specific subunit of the PBAF complex. Additionally, we identified other functionally relevant interacting partners, such as the protein arginine methyltransferase PRMT5, the E3 ubiquitin ligase substrate recognition component FBXO22, and SMARCA5 (SNF2H), a component of the ISWI complex, within the BACH1-containing protein complex (Fig. 3A, upper panel). The association between BACH1 and the SWI/SNF complex was further validated by WB analysis of eluates obtained from the FLAG-M2 affinity purification in HEK 293 T cells (Fig. 3A, lower panel). A comprehensive list of all proteins identified by mass spectrometry is provided in Supplementary Table 6.

To further validate these findings, we performed co-immunoprecipitation (Co-IP) experiments using whole cell lysates from HEK 293 T cells. Immunoprecipitation (IP) with antibodies against BACH1, followed by immunoblotting (IB) with antibodies against BRG1, PBRM1, and ARID1A representing the SWI/SNF complex, demonstrated that BACH1 was co-immunoprecipitated with the SWI/SNF complex. Reciprocally, IP with antibodies against BRG1, PBRM1, and ARID1A, followed by IB with antibodies against BACH1, confirmed that the core subunits of the SWI/SNF complex were efficiently co-immunoprecipitated with BACH1 (Fig. 3B). The association between BACH1 and the SWI/SNF complex was also detected in FaDu and CAL 27 cells, both of which are HPV-negative HNSCC cell lines.

BRG1 is the essential ATPase module of the SWI/SNF complex. To identify the specific BACH1 domain responsible for its interaction with BRG1, we performed a series of Co-IP assays. We exogenously expressed an empty vector control, full-length FLAG-tagged BACH1, and three truncation mutants (ΔBTB, ΔZIP, ΔCNC) [33, 34], each lacking a specific domain, in HEK 293 T cells. IP was carried out using antibodies against BRG1, followed by IB with antibodies against FLAG. The results demonstrated that only the ΔBTB truncation mutant failed to interact effectively with BRG1 (Fig. 3C). Collectively, these findings indicate that BACH1 is associated with the SWI/SNF complex through its BTB domain.

### Identification of genome-wide transcription targets of the BACH1/SWI/SNF complex

**Fig. 4:**
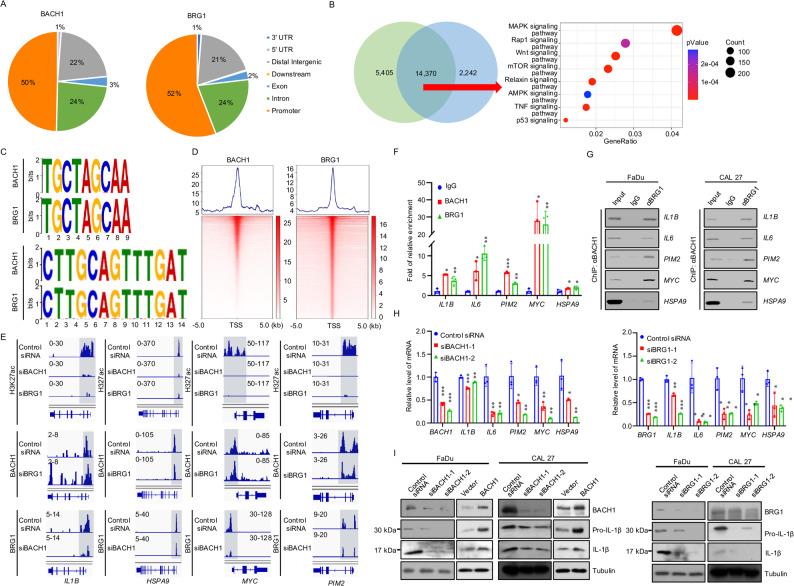
Genome-wide transcription targets analysis for the BACH1/SWI/SNF complex. **A** Genome-wide binding sites of BACH1 and BRG1 in FaDu cells determined by Cleavage Under Targets and Tagmentation (CUT&Tag) analysis. **B** Venn diagram showing the overlap of promoter regions bound by BACH1 and BRG1, with the total number of overlapping targets indicated (left). KEGG pathway enrichment analysis of the overlapping genes highlights key pathways involved in tumor biology (right). **C** Motif enrichment analysis using MEME identified conserved DNA-binding motifs in BACH1- and BRG1-bound regions. **D** CUT&Tag density heatmaps and profiles of BRG1 on BACH1-binding sites. **E** Binding profiles of BRG1, BACH1, and H3K27ac on the representative genes *IL1B*, *MYC*, *PIM2*, and *HSPA9* following knockdown of BACH1 or BRG1. **F** Quantitative ChIP (qChIP) verification of the CUT&Tag results on the promoter of the indicated genes with antibodies against BACH1 and BRG1 in FaDu cells. Results are presented as fold of change over control IgG. Error bars, mean ± SD for three independent experiments. (^*^p < 0.05, ^**^p < 0.01, ^***^p < 0.001). (G) ChIP and Re-ChIP experiments in FaDu cells with the indicated antibodies. (H) Quantitative PCR (qPCR) analysis of gene expression following siRNA-mediated knockdown of BACH1 or BRG1 in FaDu cells. Error bars, mean ± SD for three independent experiments. (^*^p < 0.05, ^**^p < 0.01, ^***^p < 0.001). (I) WB analyses of the expression of Pro-IL-1β and IL-1β in FaDu and CAL 27 cells under knockdown of BACH1 and BRG1 with different sets of small interfering RNA (siRNA) or in FaDu and CAL 27 cells transfected with the expression plasmids for BACH1

To comprehensively characterize the functional significance of the physical interaction between BACH1 and the SWI/SNF complex in HPV-negative HNSCC cells, firstly, we analyzed the genome-wide transcriptional targets of the BACH1/SWI/SNF complex using Cleavage Under Targets and Tagmentation (CUT&Tag). We performed CUT&Tag in FaDu cells, utilizing validated antibodies against BACH1 and BRG1. High-resolution chromatin profiling data was obtained, and the data were analyzed through Model-based Analysis of ChIP-seq version 14 (MACS14) with a stringent statistical threshold (q-value < 0.05), identifying 19,775 significant BACH1 binding loci and 16,612 BRG1-associated chromatin regions genome-wide (Fig. 4A). Genomic annotation of these binding sites revealed that approximately 50% of BACH1 peaks and 52% of BRG1 peaks were localized within promoter regions, suggesting their predominant role in transcriptional regulation (Fig. 4A). Integrative bioinformatics analysis identified 14,370 genes exhibiting co-occupancy by both BACH1 and BRG1 at their promoter regions, defining these as co-regulated transcriptional targets of the BACH1/SWI/SNF complex. Kyoto Encyclopedia of Genes and Genomes (KEGG) pathway enrichment analysis of these co-targeted genes revealed significant enrichment for multiple cancer-relevant pathways, including Wnt signaling pathway, mTOR signaling pathway, and p53 signaling pathway (Fig. 4B). De novo motif discovery analysis using MEME-ChIP revealed highly similar sequence specificity between BACH1 and BRG1, with both factors preferentially recognizing a core consensus sequence, such as TGCTAGCAA and CTTGCAGTTTGAT, a canonical MARE motif (Fig. 4C). Importantly, genomic landscape analysis showed that BRG1 was significantly enriched in regions surrounding BACH1 binding sites (Fig. 4D).

To elucidate the directionality of recruitment between BACH1 and the SWI/SNF complex, we further performed CUT&Tag assays in FaDu cells following knockdown of BACH1 or BRG1, using antibodies against BRG1, BACH1, and the active chromatin mark H3K27ac. Integrated analysis with our previous datasets revealed that BACH1 depletion significantly reduced the binding levels of both BRG1 and H3K27ac at the promoters of target genes (such as *IL1B*, *HSPA9*, *MYC*, and *PIM2*), as shown in the IGV tracks (Fig. 4E). Upon BRG1 knockdown, while H3K27ac signals at these loci decreased, BACH1 binding capacity remained largely unaffected (Fig. 4E). Taken together, these results demonstrate that BACH1 recruits the SWI/SNF complex, which subsequently facilitates the establishment of active chromatin to drive transcription. Detailed CUT&Tag results are provided in Supplementary Table 7.

To validate the CUT&Tag results, we performed quantitative chromatin immunoprecipitation (qChIP) experiments in FaDu cells using specific antibodies against BACH1 and BRG1, targeting various genes. The results showed that both BACH1 and BRG1 were bound to the promoters of co-targets genes, such as *IL1B*, *IL6*, *PIM2*, *MYC*, and *HSPA9* (Fig. 4F). To further support the hypothesis that BACH1 recruits the SWI/SNF complex to form a functional protein complex for regulating the expression of target genes, ChIP/Re-ChIP assays were performed on these representative genes. Soluble chromatin was initially immunoprecipitated using antibodies against BACH1, and the eluates from the first immunoprecipitation were subsequently re-immunoprecipitated using antibodies against BRG1. We found that promoters of *IL1B*, *IL6*, *PIM2*, *MYC*, and *HSPA9*, initially immunoprecipitated with antibodies against BACH1, could also be re-immunoprecipitated with antibodies against BRG1 in both FaDu and CAL 27 cells (Fig. 4G). Consistent with a functional role for these complexes, knockdown of BACH1 or BRG1 reduced the expression of all the target genes (Fig. 4H). Furthermore, we knocked down BACH1 or BRG1 in FaDu cells and performed WB. The results showed that loss of BACH1 or BRG1 function was associated with reduced expression of both pro-IL-1β (31 kDa precursor) and cleaved IL-1β (17 kDa mature form) (Fig. 4I). Furthermore, both the precursor and mature forms of IL-1β were elevated in response to increased BACH1 expression (Fig. 4I). These results support the coexistence of BACH1 and the SWI/SNF complex at the promoter regions of target genes.

### IL-1β serves as a key oncogenic factor in HNSCC

**Fig. 5:**
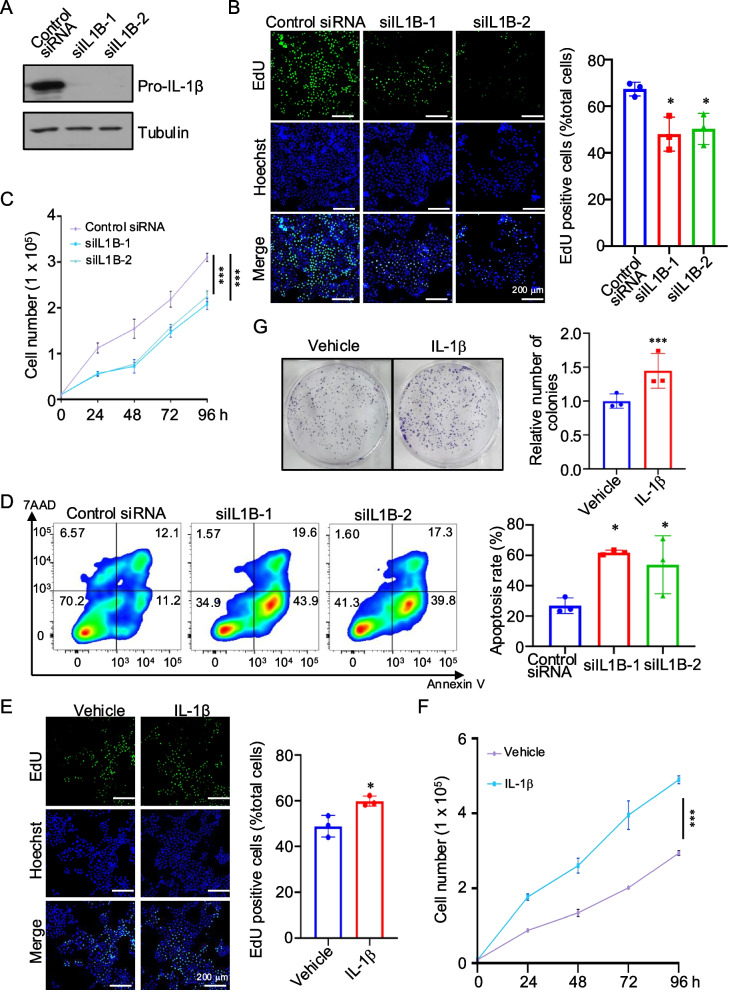
IL-1β promotes proliferation and inhibits apoptosis of cancer cells. **A** The knockdown efficiency was validated by WB. **B** EdU incorporation assay to assess DNA synthesis in IL-1β knockdown FaDu cells. Scale bar: 200 μm. Error bars, mean ± SD for three independent experiments. (^*^p < 0.05). **C** Growth curve in IL-1β-deficient FaDu cells. Error bars, mean ± SD for three independent experiments. (^***^p < 0.001). **D** Apoptosis assay in IL-1β-deficient FaDu cells. Error bars, mean ± SD for three independent experiments. (^*^p < 0.05). (E) EdU incorporation assay in FaDu cells treated with exogenous IL-1β. Scale bar: 200 μm. Error bars, mean ± SD for three independent experiments. (^*^p < 0.05, ^**^p < 0.01). **F** Growth curve of FaDu cells treated with exogenous IL-1β. Error bars, mean ± SD for three independent experiments. (^***^p < 0.001). **G** FaDu cells treated with exogenous IL-1β were cultured for 14 days, followed by crystal violet staining and colony counting. Representative images and quantification are shown. Error bars represent the mean ± SD from three independent experiments. (^***^p < 0.001)

As previously noted, abnormally high expression of IL-1β is commonly observed in various tumor types [35, 36]. IL-1β directly acts on tumor cells, activating the MAPK and PI3K/AKT signaling pathway, which enhances tumor cell proliferation, survival, and invasion, ultimately promoting tumor progression [32, 37]. To investigate the functional role of IL-1β in the progression of HNSCC, we initially knocked down IL-1β in FaDu cells using small interfering RNA (siRNA) (Fig. 5A). EdU incorporation assays revealed a significant reduction in the proliferative capacity of IL-1β-depleted cells compared to control cells (Fig. 5B). Consistent with these findings, growth curve analysis demonstrated a marked decrease in proliferation rate following IL-1β knockdown (Fig. 5C). Furthermore, flow cytometry analysis using Annexin V/7-AAD double staining showed a pronounced increase in both early and late apoptotic cell populations following IL-1β silencing (Fig. 5D), indicating that IL-1β is essential for both proliferation and survival of HNSCC cells. To assess the gain-of-function effects, we stimulated FaDu cells with recombinant human IL-1β protein. EdU incorporation assays showed an increase in the percentage of proliferating cells in the IL-1β -treated group compared to untreated controls (Fig. 5E). Growth curve analysis further confirmed that IL-1β stimulation significantly enhanced cell growth kinetics (Fig. 5F). Additionally, colony formation assays revealed a substantial increase in colony number following IL-1β treatment, suggesting a pro-proliferative and pro-survival function of IL-1β (Fig. 5G). Collectively, these data suggest that IL-1β functions as a potent oncogenic cytokine that promotes proliferation and inhibits apoptosis of HNSCC cells.

### BACH1 executes its oncogenic role by driving an IL-1β-mediated autocrine/paracrine loop

**Fig. 6:**
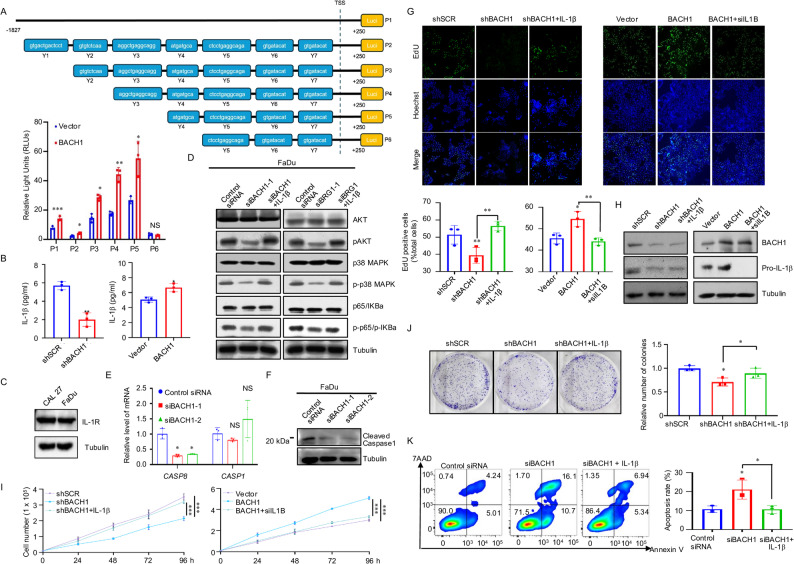
BACH1 executes its oncogenic role by driving an IL-1β-mediated autocrine or paracrine loop. **A** Schematic of the *IL1B* promoter illustrating the BACH1-binding region identified by CUT&Tag analysis and the corresponding luciferase reporter constructs. Relative luciferase activity of *IL1B* promoter deletion constructs with or without BACH1 overexpression. Error bars represent the mean ± SD from three independent experiments. (^*^p < 0.05, ^**^p < 0.01, ^***^p < 0.001). **B** Measurement of IL-1β secretion by ELISA upon BACH1 overexpression or knockdown. Error bars represent the mean ± SD from three independent experiments. (^*^p < 0.05, ^**^p < 0.01). **C** WB analysis of IL-1R expression in CAL 27 and FaDu cells. **D** WB analysis of IL-1R signaling effectors in FaDu cells with knockdown of BACH1 or BRG1, treated with or without exogenous IL-1β. **E** qPCR analysis of gene expression following knockdown of BACH1 in FaDu cells. Error bars, mean ± SD for three independent experiments. (^*^p < 0.05). (F) WB analysis of cleaved-Caspase1 expression in FaDu cells with BACH1 knockdown. **G** EdU incorporation assay in FaDu cells with BACH1 knockdown and exogenous IL-1β, or stable BACH1 overexpression transfected with IL-1β siRNA. Scale bar: 200 μm. Error bars represent the mean ± SD from three independent experiments. (^*^p < 0.05, ^**^p < 0.01). **H** WB analysis validating the knockdown or expression efficiency in FaDu cells. (I) Growth curve of FaDu cells with BACH1 knockdown and exogenous IL-1β, or stable BACH1 overexpression transfected with IL-1β siRNA. Error bars, mean ± SD for three independent experiments. (^***^p < 0.001). (J) FaDu cells treated with BACH1 knockdown and exogenous IL-1β, or stable BACH1 overexpression transfected with IL-1β siRNA, were cultured for 14 days, followed by crystal violet staining and colony counting. Representative images and quantification are shown. Error bars represent the mean ± SD from three independent experiments. (^*^p < 0.05). (K) Apoptosis assay in FaDu cells treated with BACH1 knockdown and exogenous IL-1β, or stable BACH1 overexpression transfected with IL-1β siRNA. Error bars, mean ± SD for three independent experiments. (^*^p < 0.05)

To functionally validate that BACH1 is the upstream mechanism driving aberrant *IL1B* expression, we first observed a prominent BACH1 binding peak located −1827—+ 250 bp of the *IL1B* transcriptional start site in CUT&Tag analysis (P1). Although this region lacks the canonical MARE motif typically associated with BACH1 transcription, it contains the Y1-7 binding motifs (P2-6). We functionally validated BACH1-dependent transcriptional activity using a luciferase reporter system in HeLa cells containing a fragment harboring these motifs. These experiments confirmed that the Y4 motif in the *IL1B* promoter constitutes a functional BACH1-binding region (Fig. 6A).

To confirm that secreted IL-1β can act on tumor cells in an autocrine or paracrine manner, we first demonstrated that BACH1 knockdown significantly reduced IL-1β secretion into the culture medium. Conversely, BACH1 overexpression induced a higher level of IL-1β secretion into the cell culture supernatant (Fig. 6B). We then demonstrated that IL1R1 expression in CAL 27 and FaDu cells (Fig. 6C). Furthermore, the deficiency of BACH1 or BRG1 significantly reduced the phosphorylation levels of key effectors, including AKT, p38 MAPK, and NF-kB/p65, suggesting that the absence of BACH1 or BRG1 leads to the impairment of the IL-1–IL-1R signaling pathway (Fig. 6D). Notably, the addition of exogenous, mature IL-1β partially restored this signaling pathway (Fig. 6D). The mature form of IL-1β is produced by Caspase-1-mediated cleavage of its precursor, pro-IL-1β. Caspase-1 activation itself occurs through auto-cleavage or cleavage by Caspase-8 [38, 39]. While CUT&Tag analysis did not identify *CASP1* as a direct BACH1 target, it did transcriptionally upregulate *CASP8*, a finding validated by qPCR (Fig. 6E). Consistent with this, BACH1 knockdown reduced the levels of pro-IL-1β, its cleaved active form (mature IL-1β), and the cleaved fragment of Caspase-1 (Figs. 4I and 6F). Collectively, these results position BACH1 as an upstream driver that orchestrates aberrant IL-1β production and activation.

To further establish whether the oncogenic phenotype of BACH1 is mediated by IL-1β, we performed stringent rescue experiments. The addition of exogenous IL-1β to BACH1-knockdown cells partially restored their proliferation, colony-forming capacity, and reduced apoptosis (Figs. 6G-K). Conversely, knockdown of IL1B in BACH1-overexpressing cells reversed their hyper-proliferative phenotype (Figs. 6G-K). These findings directly demonstrate that IL-1β is a critical downstream effector of BACH1. Together, these results support a coherent model in which BACH1 drives both the production and processing of IL-1β, at least in part through direct transcriptional activation of *Caspase8*. The secreted mature IL-1β then activates proliferative signaling pathways (such as AKT or NF-kB) within tumor cells in an autocrine/paracrine manner via IL-1R.

### BACH1 orchestrates IL-1β production and maturation via the PBAF complex

**Fig. 7:**
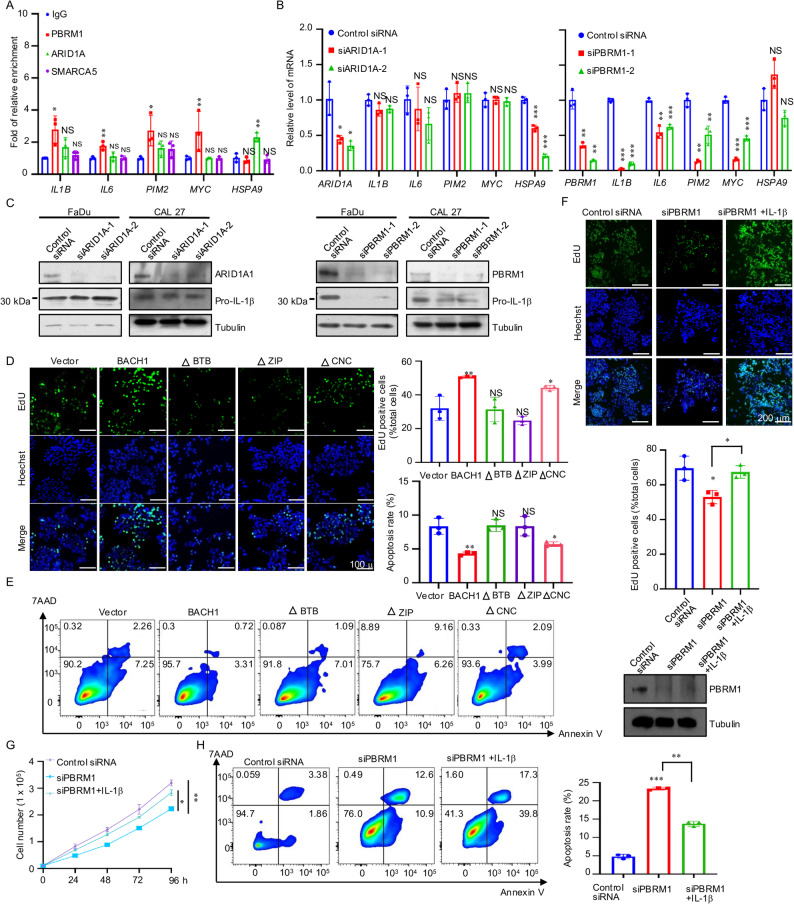
BACH1 orchestrates IL-1β production and maturation via the PBAF complex. **A** qChIP analysis of PBRM1, ARID1A, and SMARCA5 binding at the promoters of indicated genes in FaDu cells. Results are presented as fold of change over control IgG. Error bars, mean ± SD for three independent experiments. (^*^p < 0.05, ^**^p < 0.01). **B** qPCR analysis of gene expression following knockdown of PRBM1 or ARID1A in FaDu cells. Error bars, mean ± SD for three independent experiments. (^*^p < 0.05, ^**^p < 0.01, ^***^p < 0.001). **C** WB analysis of pro-IL-1β expression in FaDu cells with ARID1A or PBRM1 knockdown. (D-E) EdU incorporation assay and apoptosis assay of FaDu cells overexpressing wild-type BACH1 or its deletion mutants. Error bars, mean ± SD for three independent experiments. (^*^p < 0.05, **p < 0.01). (F–H) EdU incorporation assay, growth curve, and apoptosis assay in FaDu cells following PBRM1 knockdown with or without exogenous IL-1β treatment. Scale bar: 200 μm. Error bars represent the mean ± SD from three independent experiments. (^*^p < 0.05, **p < 0.01, ***p < 0.001)

Given that our mass spectrometry analysis revealed that BACH1 is associated with components of the cBAF, PBAF, and ISWI chromatin remodeling complexes, we sought to determine which specific complex mediates BACH1-driven transcription. We performed qChIP assays in FaDu cells using antibodies against ARID1A (cBAF), PBRM1 (PBAF), and SMARCA5 (ISWI). The results revealed distinct subcomplex recruitment: ARID1A was enriched at the *HSPA9* promoter, while PBRM1 bound to promoters of *IL1B*, *IL6*, *PIM2*, and *MYC*. SMARCA5 showed no significant binding to these targets (Fig. 7A). Importantly, the transcriptional effects were subcomplex-specific: ARID1A knockdown specifically decreased HSPA9 expression, whereas PBRM1 depletion reduced expression of IL1B, IL6, PIM2, and MYC (Fig. 7B). Furthermore, we knocked down ARID1A or PBRM1 in FaDu cells and performed WB. The results showed that loss of PBRM1 function was associated with reduced expression of pro-IL-1β, whereas ARID1A depletion had no effect on IL1β expression (Fig. 7C). Similar results were obtained in CAL 27 cells (Fig. 7C). Together, these results establish that BACH1 recruits distinct SWI/SNF subcomplexes to activate transcription of the respective target genes.

Further investigation revealed that the loss-of-function mutants ΔBTB and ΔZIP both lost the ability to promote cell proliferation and inhibit apoptosis (Fig. 7D-E). Specifically, the functional failure of ΔZIP stems from the loss of its DNA-binding capacity. In contrast, the results for ΔBTB reveal that the pro-oncogenic function of BACH1 is highly dependent on the interaction between this domain and BRG1, the core ATPase of the SWI/SNF complex. To further identify the specific chromatin remodeling subcomplex recruited by BACH1, we performed knockdown assays for ARID1A (cBAF), PBRM1 (PBAF), and SMARCA5 (ISWI). The results showed that silencing any of these factors significantly inhibited cell growth and induced apoptosis (Fig. 7F-H, Supplementary Fig. 2). Crucially, a rescue experiment demonstrated that the supplementation of exogenous IL-1β could only specifically rescue the phenotypic defects caused by PBRM1 deficiency (Fig. 7F-H, Supplementary Fig. 2). This finding strongly demonstrates that the PBAF subcomplex is the core factor mediating this biological effect.

### The BACH1-IL-1β axis reprograms the transcriptome and amplifies its signal through a positive feedback loop

**Fig. 8:**
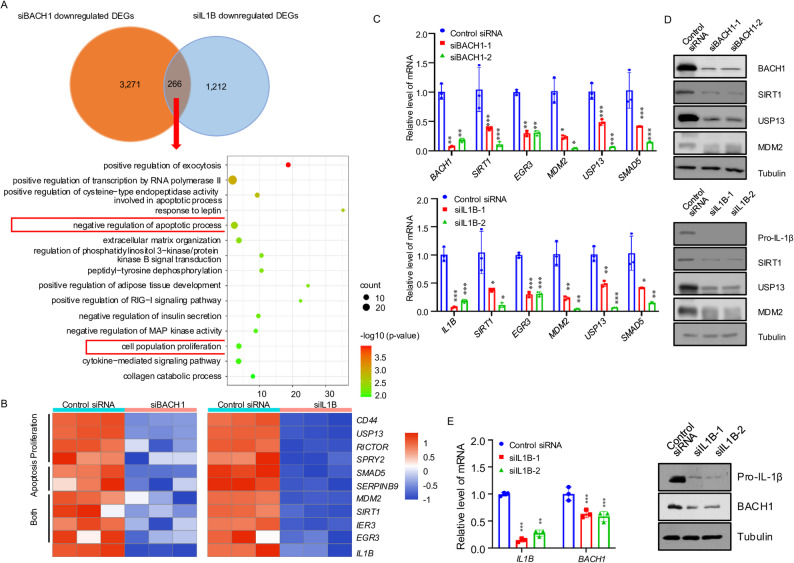
The BACH1-IL-1β axis reprograms the transcriptome and amplifies its signal through a positive feedback loop. **A** Venn diagram illustrating the overlap of downregulated genes between RNA-sequencing (RNA-seq) of FaDu cells transfected with IL-1β siRNA and BACH1 siRNA. The numbers indicate the count of downregulated genes (upper). Gene Ontology (GO) pathway enrichment analysis of the overlapping downregulated genes, showing the top pathways enriched by these genes (lower). **B** The heatmap of downregulated gene expression from RNA-seq of FaDu cells transfected with BACH1 siRNA and IL-1β siRNA. Each row is a gene, and the color shows the low (blue) to high (orange) gene expression levels. **C** qPCR verification of gene expression from RNA-seq following knockdown of BACH1 or IL1B in FaDu cells, showing the expression levels of *BACH1*, *IL1B*, *SIRT1*, *EGR3*, *MDM2*, *USP13*, and *SMAD5*. Error bars represent the mean ± SD from three independent experiments. (^*^p < 0.05, ^**^p < 0.01, ^***^p < 0.001). **D** WB validation of the expression of the indicated protein in FaDu cells under knockdown of BACH1 or IL-1β with siRNA. **E** qPCR and WB analyses of the expression of BACH1 in IL-1β-deficient FaDu cells. Error bars represent the mean ± SD from three independent experiments. (^**^p < 0.01, ^***^p < 0.001)

To better understand the molecular mechanisms underlying the promotion of HNSCC progression by the BACH1-IL-1β axis, we performed RNA sequencing (RNA-seq) to analyze transcriptome changes following the knockdown of BACH1 and IL-1β. Using a q value cutoff of 0.05, we identified 3,537 genes that were downregulated upon BACH1 knockdown and 1,478 genes that were downregulated following IL-1β loss-of-function (Fig. 8A). Cross-analysis of these two gene sets revealed 266 genes that were downregulated in both groups, suggesting that these genes represent downstream targets regulated by BACH1-IL-1β axis (Fig. 8A). Gene ontology (GO) functional enrichment analysis of these genes revealed significant enrichment in biological processes such as the negative regulation of apoptosis and promotion of cell proliferation (Fig. 8A). To further investigate the differential expression of genes within these signaling pathways, we analyzed the expression profiles of several key regulatory genes, including *USP13* [40], *CD44* [41], *SPRY2* [42], and *RICTOR* [43], which are primarily associated with the positive regulation of cellular proliferation; *SERPINB9* [44], *SMAD5* [45], both of which have been implicated in the negative regulation of cell apoptosis; as well as *MDM2* [46], *SIRT1* [47, 48], *IER3* [49], *EGR3* [50], all of them simultaneously promote proliferation and inhibit apoptosis. The results indicated that, upon knockdown of either BACH1 or IL-1β, the expression of these representative genes was significantly reduced (Fig. 8B). These findings suggest that the BACH1-IL-1β axis plays a crucial role in tumor progression by modulating cell survival and proliferation through transcriptional reprogramming. Detailed RNA-seq results are provided in Supplementary Table 8. The raw data have been uploaded to the GEO database, with the accession number GSE325366.

Furthermore, we strategically selected *SIRT1, EGR3*, *MDM2*, *USP13*, and *SMAD5* as genes linked to proliferation and apoptosis regulation. qPCR and WB analyses revealed that in FaDu cells, knockdown of BACH1 or IL-1β led to a significant downregulation of the mRNA levels of these genes, consistent with the RNA-seq results (Fig. 8C), as well as a reduction in the protein level of SIRT1, USP13 and MDM2 (Fig. 8D). Notably, in the RNA-seq analysis of IL-1β knockdown, we found that *BACH1* was also downregulated, suggesting the existence of a BACH1-IL-1β positive feedback loop in cells. To further validate this hypothesis, we measured the BACH1 expression following IL-1β knockdown. As anticipated, BACH1 expression was downregulated at both the mRNA and protein levels after the IL-1β knockdown (Fig. 8E). These results collectively suggest that BACH1-IL-1β forms a feedback loop that inhibits apoptosis and promotes proliferation via transcriptional reprogramming of downstream genes.

### Interleukin-1 receptor antagonist Anakinra blocks HPV-negative HNSCC progression by targeting BACH1-IL-1β

**Fig. 9:**
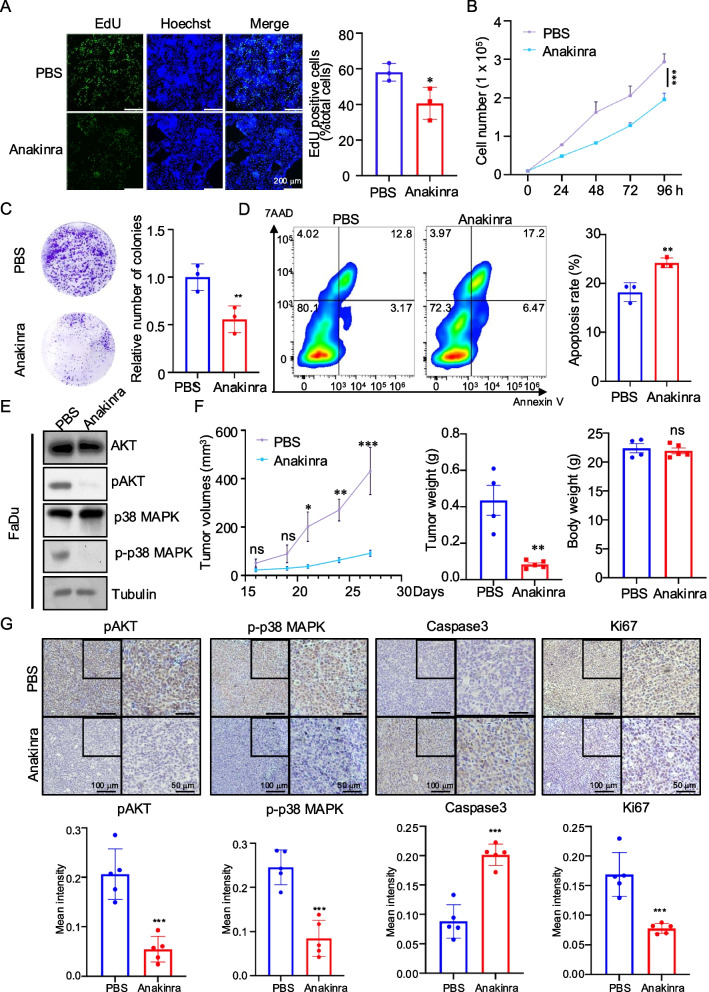
Interleukin-1 receptor antagonist Anakinra blocks HPV-negative HNSCC progression by targeting BACH1-IL-1β **A** EdU incorporation assay in FaDu cells treated with PBS or Anakinra. Scale bar: 200 μm. Error bars represent the mean ± SD from three independent experiments. (^*^p < 0.05). **B** Growth curve of FaDu cells treated with PBS or Anakinra. Error bars, mean ± SD for three independent experiments. (^***^p < 0.001). **C** FaDu cells treated with PBS or Anakinra were cultured for 14 days, followed by crystal violet staining and colony counting. Representative images and quantification are shown. Error bars represent the mean ± SD from three independent experiments. (^**^p < 0.01). **D** Apoptosis assay in FaDu cells treated with PBS or Anakinra. Error bars, mean ± SD for three independent experiments. (^**^p < 0.01). **E** WB analyses of the expression of AKT, phospho-AKT (p-AKT), p38 MAPK and p-p38 MAPK in FaDu cells treated with or without Anakinra. **F** In xenograft-bearing female athymic mice, treatment with PBS or Anakinra was initiated after tumors reached 50–100 mm.^3^. Tumor volume (left), tumor weight (middle), and body weight of mice (right) were measured and monitored throughout the treatment period. PBS group, *n* = 4; Anakinra group, *n* = 5. Error bars represent the mean ± SEM from independent animals (*p < 0.05, **p < 0.01, ***p < 0.001). **G** Representative IHC staining images of pAKT, p-p38 MAPK, Caspase3, and Ki67 in FFPE tumor tissues from the PBS group (*n* = 4) and Anakinra group (*n* = 5). The average staining intensity was quantified using Image-Pro Plus software. Each dot represents a tumor sample from one mouse. Error bars indicate mean ± SEM (***p < 0.001)

To address the limited therapeutic efficacy of Hemin, despite its established role as a natural BACH1 inhibitor, we explored alternative strategies targeting the newly identified BACH1-IL-1β regulatory axis in HPV-negative HNSCC. Based on our findings demonstrating the critical role of this feedback loop in tumor progression, we hypothesized that pharmacological inhibition of the downstream cytokine IL-1β could provide a more effective therapeutic approach. We therefore evaluated the anti-tumor potential of Anakinra, a clinically approved IL-1 receptor antagonist, both in vitro and in vivo. EdU incorporation assays demonstrated a significant reduction in proliferative capacity in Anakinra-treated cells compared to vehicle-treated group (Fig. 9A). Consistent with this result, growth curve analysis confirmed a substantial inhibition of cell growth in the presence of Anakinra (Fig. 9B). Furthermore, colony formation assays revealed a marked decrease in the number of colonies following Anakinra treatment (Fig. 9C). Flow cytometry, using Annexin V/7-AAD staining, showed a significant increase in both early and late apoptotic populations in Anakinra-treated cells (Fig. 9D). Moreover, we treated FaDu cells with Anakinra and assessed the activation status of key downstream pathways of the IL-1 receptor by measuring the phosphorylation levels of AKT and p38 MAPK via WB. Our results demonstrate that phosphorylation of these signaling effectors was significantly reduced following Anakinra treatment, indicating effective blockade of IL-1β/IL-1R axis signaling (Fig. 9E). These data collectively indicate that Anakinra suppresses proliferation and promotes apoptosis of tumor cells in vitro.

To validate the tumor suppressive effect of Anakinra in vivo, we established a xenograft model by subcutaneously implanting FaDu cells into the 6–8 weeks old athymic nude mice. Mice were randomized to receive either PBS or Anakinra after tumors reached 50–100 mm^3^. Tumor volume and weight measurements showed a significant reduction in the Anakinra-treated group compared to control, while body weights remained stable throughout the treatment period, indicating minimal systemic toxicity (Fig. 9F). Mechanistically, immunohistochemistry analysis of tumor tissues revealed that Anakinra treatment effectively suppressed the IL-1β signaling axis, as shown by reduced the phosphorylation of AKT and p38 MAPK (Fig. 9G). Concurrently, decreased Ki-67 and increased cleaved Caspase-3 expression demonstrated inhibited proliferation and induced apoptosis (Fig. 9G). These consistent anti-tumor effects across experimental models provide compelling preclinical evidence that IL-1β blockade via Anakinra could represent a viable therapeutic strategy for HPV-negative HNSCC patients, particularly those with activated BACH1-IL-1β signaling axis.

### Clinicopathological relevance of the BACH1/SWI/SNF-IL-1β axis in HNSCC

**Fig. 10:**
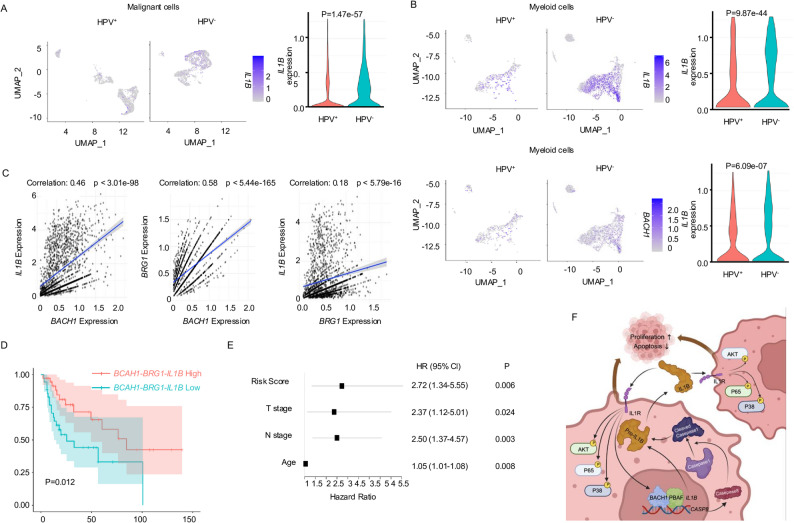
Clinicopathological relevance of the BACH1/SWI/SNF-IL-1β axis in HNSCC. **A** and **B** Expression of *IL1B* and BACH1 in malignant or myeloid cells from HPV-positive and HPV-negative HNSCC, derived from the publicly available scRNA-seq dataset (GSE181919). *IL1B* and HPV expression is visualized using UMAP and quantified by violin plots. **C** Correlation analysis of gene expression: Correlation between *BACH1* and *IL1B* expression (left). Correlation between *BACH1* and *BRG1* expression (middle). Correlation between *IL1B* and *BRG1* expression (right). **D** Kaplan–Meier survival analysis in HPV-negative HNSCC based on gene signatures formed from *BACH1*, *SMARCA4*, and *IL1B* in the TCGA database. **E** A multivariate Cox regression analysis using the TCGA-HNSC cohort. **F** The proposed model for the BACH1/SWI/SNF-IL-1β axis in HNSCC

To evaluate the clinical relevance of the BACH1/SWI/SNF–IL-1β axis, we further analyzed the publicly available scRNA-seq dataset (GSE181919). We found that within the malignant epithelial subpopulation, IL1B expression was also elevated in HPV-negative tumors (Fig. 10A). Notably, myeloid immune cells from HPV-negative patients exhibited significantly higher expression of BACH1 and IL1B than those from HPV-positive patients (Fig. 10B). Furthermore, the presence of a coordinated regulatory network was supported by a significant positive correlation between the expression levels of *BACH1*, *BRG1*, and *IL1B* in HPV-negative HNSCC samples (Fig. 10C). To further evaluate the clinical significance of this axis, we found that a BACH1/BRG1/IL1B expression signature was associated with poor patient prognosis (Fig. 10D). Multivariate Cox regression analysis of the TCGA-HNSC cohort, adjusted for T stage, N stage, and age, revealed that this combined expression signature serves as an independent risk factor for unfavorable outcomes (Figs. 10E). Collectively, these findings suggest that the BACH1/SWI/SNF-IL-1β regulatory axis plays a crucial role in the malignant progression of HPV-negative HNSCC and could serve as a potential marker for evaluating patient disease stratification (Fig. 10F).

## Discussion

HPV-negative head and neck squamous cell carcinoma (HNSCC) exhibits significantly greater biological aggressiveness and clinical heterogeneity compared to its HPV-positive counterpart. This malignancy is characterized by higher incidence rates, more limited therapeutic options, and poorer treatment outcomes [2–5]. These clinical differences underscore the urgent need to better understand the molecular mechanisms that drive regulation in HPV-negative HNSCC. In this study, we identify BACH1 as a pivotal oncogenic transcriptional regulator in HPV-negative HNSCC. Mechanistically, BACH1 transcriptionally activates *IL1B* expression by recruiting the SWI/SNF PBAF chromatin remodeling complex, promoting proliferation and inhibiting apoptosis of tumor cells through extensive downstream transcriptional reprogramming processes. Importantly, silencing IL-1β results in a reduction in BACH1 expression, suggesting the presence of a self-reinforcing positive feedback loop that accelerates tumor progression in HPV-negative HNSCC. Pharmacologic inhibition of this loop using the interleukin-1 receptor (IL-1R) antagonist Anakinra effectively suppresses tumor progression, highlighting the therapeutic potential of targeting the BACH1-IL-1β loop. High expression of the BACH1/SWI/SNF-IL-1β loop is associated with poor patient prognosis, positioning this loop as both a prognostic biomarker and a promising therapeutic target. While these findings provide important mechanistic insights, there are limitations that must be considered. The tumor type-specificity of this regulatory loop remains to be fully elucidated, and further investigation is needed to determine whether the BACH1-IL-1β loop operates in other malignancies or is a feature unique to HPV-negative HNSCC. Additionally, bioinformatic analysis of The Human Protein Atlas reveals notable BACH1 expression in normal tissues, including bone marrow, placenta, and liver. This raises questions about whether this regulatory circuit exists and has a potential physiological role in development and homeostasis, which warrants further exploration.

As a member of the BTB-bZIP transcription factor family, BACH1 exhibits oncogenic properties through its dysregulated expression and aberrant subcellular localization in multiple malignancies, including breast and colorectal carcinomas, and promotes tumorigenesis by regulating cellular oxidative stress responses [14, 51]. Our findings establish that high expression of BACH1 in HPV-negative HNSCC leads to the upregulation of genes that inhibit apoptosis and promote proliferation, ultimately driving tumor progression. However, several critical mechanistic questions remain unresolved: First, while BACH1's nuclear translocation is known to be dynamically regulated by environmental stimuli, the specific molecular determinants that facilitate its nuclear accumulation in HPV-negative HNSCC require systematic investigation. Second, although BACH1 represents a promising therapeutic target, our experimental data demonstrate that pharmacological intervention with Hemin, a physiological BACH1 inhibitor, fails to elicit significant antitumor effects. Notably, while Hemin administration successfully induced heme oxygenase-1 (HO-1) expression in renal tissues, confirming target engagement, it did not significantly reduce intratumoral BACH1 protein levels. In the future, it will be important to elucidate the underlying mechanisms that restrict Hemin's bioavailability in tumor tissue and its inability to effectively downregulate BACH1 expression, such as investigating potential barriers to tumor penetration and cell-intrinsic resistance mechanisms that modulate Hemin sensitivity.

The SWI/SNF chromatin remodeling complex family comprises three evolutionarily conserved subtypes distinguished by their subunit composition: canonical BAF (BRG1/BRM-associated factor), PBAF (polybromo-associated BAF), and non-canonical BAF (ncBAF) complexes [52, 53]. These multi-subunit assemblies share a conserved structural core in which either BRG1 (SMARCA4) or BRM (SMARCA2) serves as the catalytic ATPase subunit, driving nucleosome repositioning via ATP-dependent chromatin remodeling. The functional specialization of each complex subtype is dictated by the incorporation of mutually exclusive subunits. For example, complexes containing ARID1A or ARID1B define the canonical BAF subtype. Of note, *ARID1A* is a well-established tumor suppressor gene [54]. Its inactivation disrupts SWI/SNF-mediated chromatin remodeling, leading to defects in DNA damage repair and transcriptional regulation, ultimately contributing to oncogenic transformation and tumor progression [54, 55]. Our current findings uncover an unexpected association between the oncogenic protein BACH1 and the tumor suppressor ARID1A within this complex. This paradoxical interaction highlights the remarkable functional plasticity of the SWI/SNF complex and suggests its dual roles in oncogenic and tumor-suppressive regulatory networks, which may be dictated by cellular context and specific interacting partners. However, the biological significance and molecular mechanisms of this dual functionality remain to be fully elucidated.

IL-1β is mainly secreted by monocytes and macrophages and promotes tumor progression by regulating multiple biological processes such as immune evasion and angiogenesis [56]. In this study, we observed that IL-1β is overexpressed in HPV-negative HNSCC compared to HPV-positive tumors, especially in malignant epithelial cell subsets, suggesting that tumor cells in HPV-negative HNSCC may also secrete IL-1β. This finding aligns with emerging evidence indicating that certain tumors establish autocrine IL-1β signaling loops to sustain oncogenic phenotypes [37]. For instance, in breast cancer, tumor-derived IL-1β promotes epithelial-mesenchymal transition (EMT) through downregulation of E-cadherin and upregulation of N-cadherin, β-catenin, and γ-catenin [56]. Similarly, gastric cancer cells exploit IL-1β to activate ERK1/2-dependent transcriptional programs involving CREB and C/EBPβ, enhancing proliferative and survival signaling [57]. While these findings support the tumor-promoting role of IL-1β, our study uniquely identifies a positive correlation between IL-1β expression and BACH1 levels in HPV-negative HNSCC. However, since IL-1β lacks nuclear translocation capacity, its regulatory effect on BACH1 expression should not rely on transcriptional regulation but is more likely mediated by intracellular signal transduction cascades, which remains to be elucidated in future studies.

Anakinra, a recombinant human interleukin-1 receptor antagonist (IL-1Ra), functions as a competitive inhibitor of both IL-1α and IL-1β by binding with high affinity to IL-1 receptor type 1 (IL-1R1), thereby effectively blocking downstream signaling pathways [58]. It is currently approved by the U.S. Food and Drug Administration (FDA) for the treatment of rheumatoid arthritis and various autoimmune disorders [59], as well as for managing cytokine release syndrome (CRS) in patients receiving CAR-T cell therapy [60, 61]. Although Anakinra has not yet been formally classified as an antineoplastic agent to date, increasing preclinical evidence supports its potential therapeutic value in oncology, owing to the central role of IL-1 signaling in modulating the tumor microenvironment. In colorectal cancer models, Anakinra has been shown to disrupt the crosstalk between cancer-associated fibroblasts and IL-17^+^ immune cells, thereby restoring antitumor immune responses [62]. It also suppresses pro-inflammatory gene signatures in both tumor cells and peripheral blood mononuclear cells, resulting in substantial inhibition of tumor migration and proliferation in glioblastoma models [63]. Additionally, Anakinra inhibits bone metastasis in breast cancer and reduces bone turnover markers such as IL-1β and TNFα, further supporting its utility in targeting bone metastasis [64]. In a phase II clinical trial involving patients with metastatic colorectal cancer, Anakinra was combined with 5-fluorouracil (5-FU) and bevacizumab (an anti-VEGF monoclonal antibody), significantly increasing the median progression-free survival (PFS) and overall survival (OS) of patients [65]. These findings suggest that Anakinra has potential synergistic effects with conventional treatment regimens. Although IL-1β has been implicated in the maintenance of cancer stem cell phenotypes [37], few studies have systematically explored Anakinra's direct effect on tumor cell-intrinsic signaling. Our study provides novel mechanistic insight, demonstrating that Anakinra exerts potent antitumor activity in HPV-negative HNSCC by directly targeting the BACH1-IL-1β feedback loop in malignant epithelial cells, rather than just regulating immunity as previously discovered, further broadening the potential clinical application of Anakinra in tumor treatment. Given the expression of IL-1β in tumor-infiltrating T cells, future studies should be utilizing immune-competent murine models to investigate the role of BACH1-IL-1β signaling in shaping the tumor immune microenvironment. Furthermore, optimized dosing regimens and administration strategies should be explored to assess whether Anakinra may synergize with immune checkpoint inhibitors, such as anti-PD-1 therapy, in the context of clinical translation.

Hemin exerted only limited inhibitory effects on BACH1 within the tumor in vivo. This stands in stark contrast to the robust induction of HO-1 and reduction of BACH1 observed in the kidney, indicating that while systemic exposure was achieved, effective intratumoral delivery and sustained drug activity were insufficient. This finding is consistent with a prior report that Hemin alone failed to suppress tumor growth in mice [66]. To understand this discrepancy, we considered the underlying biology. Under basal conditions, BACH1 forms repressive heterodimers with small MAF proteins on the *HMOX1* enhancer. Heme binding inhibits this complex's DNA-binding activity and promotes its nuclear export, thereby inducing HO-1 expression [34, 67]. In cell culture, Hemin is stable and effectively mimics endogenous heme in regulating BACH1 [10]. However, the in vivo scenario is markedly different. Free heme in circulation is rapidly sequestered by plasma proteins—first by albumin and then by hemopexin (Hpx)—forming a highly stable Hpx–heme complex that is cleared via hepatic LRP1 receptors [68, 69]. Consequently, plasma concentrations of bioactive free heme remain extremely low. We speculate that the kidney, with its high perfusion, filtration capacity, and specific tubular heme transport systems [70], can efficiently capture free Hemin, leading to strong local effects. In contrast, tumors likely lack such efficient uptake mechanisms. The combined challenges of rapid plasma protein binding, systemic hepatic clearance, and potentially poor tumor perfusion likely severely limit Hemin's intratumoral accumulation and sustained action.

Critically, the negative outcome of this experiment should not be interpreted as a failure of BACH1 as a therapeutic target. Instead, it highlights the pharmacokinetic and delivery limitations of Hemin itself. Future studies should explore more potent BACH1 degraders or employ combinatorial strategies to enhance tumor-specific delivery—for instance, by engineering analogs with reduced plasma protein binding, incorporating active targeting moieties, or utilizing nanocarrier systems to improve tumor penetration and retention. Such optimized approaches will be essential for accurately evaluating the therapeutic potential of targeting BACH1.

In summary, our study establishes that BACH1 is significantly upregulated in HPV-negative HNSCC and activates oncogenic targets (including *IL1B*) by recruiting the SWI/SNF chromatin remodeling complex, thereby reprogramming the transcriptional program to initiate pro-proliferative and anti-apoptotic, ultimately driving tumor malignant progression. Importantly, we find that the increase in IL-1β triggers the upregulation of BACH1 expression, creating a self-reinforcing oncogenic circuit. While direct pharmacological targeting of BACH1 using its natural degrader, Hemin, is not ideal, inhibition of IL-1β signaling with the FDA-approved IL-1 receptor antagonist Anakinra demonstrates significant antitumor efficacy in vitro and in vivo. Clinical correlation analyses further reveal that elevated expression of the BACH1/BRG1-IL1β axis is strongly associated with poor prognosis in patients with HPV-negative HNSCC. Collectively, these findings provide a compelling molecular framework for risk stratification and offer strong preclinical justification for pursuing IL-1β pathway inhibition as a targeted therapeutic strategy in HPV-negative HNSCC.

### Limitation of the study

While our study provides valuable mechanistic insights and comprehensive sequencing data, several limitations should be acknowledged. First, the cellular models relied primarily on a limited panel of established HPV-negative HNSCC cell lines, notably FaDu and CAL 27. Although these lines are standard models, their findings may not fully capture the genetic and phenotypic diversity present in patient tumors, potentially limiting the generalizability of our conclusions. Second, all in vivo experiments were conducted in immunodeficient mouse models. The absence of a functional immune system precludes the evaluation of critical interactions between the BACH1-IL-1β axis and the tumor microenvironment, particularly its effects on immune cell recruitment and activation, which are central to cancer progression and therapy. Finally, our analyses did not include a systematic assessment of intratumoral heterogeneity. The role of the BACH1/BRG1-IL-1β axis may vary across different subclonal populations within a tumor, which could influence treatment response and prognosis. Addressing these limitations in future work—through the use of broader cellular models, immunocompetent systems, and single-cell or spatial transcriptomic profiling of patient samples—will be crucial for translating these findings into clinically actionable strategies.

## Supplementary Information

Supplementary Material 1: Supplemental Figure 1, related to Figure 6. BACH1 executes its oncogenic role by driving an IL-1β-mediated autocrine/paracrine loop.Additional results from two independent replicates of the EdU incorporation assay in FaDu cells: comparison between the group with BACH1 knockdown plus exogenous IL-1β treatment and the group with stable BACH1 overexpression transfected with IL-1β siRNA.

Supplementary Material 2: Supplemental Figure 2, related to Figure 7. BACH1 orchestrates IL-1β production and maturation via the PBAF complex.EdU incorporation assay, growth curve, and apoptosis assay in FaDu cells following ARID1A knockdown treated with or without exogenous IL-1β. Scale bar: 200 μm. Error bars represent the mean ± SD from three independent experiments..EdU incorporation assay, growth curve, and apoptosis assay in FaDu cells following SMARCA5 knockdown treated with or without exogenous IL-1 β. Scale bar: 200 μm. Error bars represent the mean ± SD from three independent experiments.

Supplementary Material 3: Supplemental Figure 3, related to Figure 9. Interleukin-1 receptor antagonist Anakinra blocks HPV-negative HNSCC progression by targeting BACH1-IL-1β The colony formation assay performed in FaDu cells treated with PBS or Anakinra, shown as three independent replicates.

Supplementary Material 4.

Supplementary Material 5.

Supplementary Material 6.

Supplementary Material 7.

Supplementary Material 8.

Supplementary Material 9.

Supplementary Material 10.

## Data Availability

Data is provided within the manuscript or supplementary information files.
