# Supplementary material for: A positive feedback loop between BACH1 and IL-1β promotes the progression of HPV-negative head and neck squamous cell carcinoma
Source: Cell Commun Signal. 2026 May 25;24:409. doi: 10.1186/s12964-026-02957-2 (PMC13377829; doi:10.1186/s12964-026-02957-2)
Supplement: Supplementary file 1 — Supplementary Material 1: Supplemental Figure 1, related to Figure 6. BACH1 executes its oncogenic role by driving an IL-1β-mediated autocrine/paracrine loop.Additional results from two independent replicates of the EdU incorporation assay in FaDu cells: comparison between the group with BACH1 knockdown plus exogenous IL-1β treatment and the group with stable BACH1 overexpression transfected with IL-1β siRNA. [file 12964_2026_2957_MOESM1_ESM.docx]

Supplementary Figure 1

A shSCR shBACH1shBACH1+IL-1β Vector BACH1 BACH1+siIL1B

Hoechst

EdU

Merge

200



m

200



m


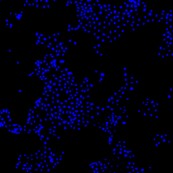

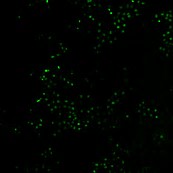

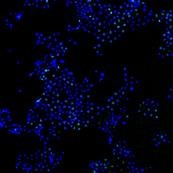

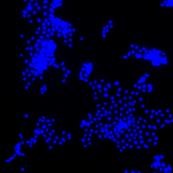

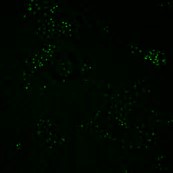

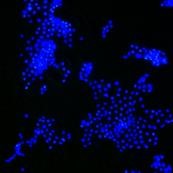

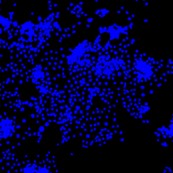

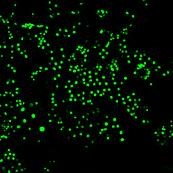

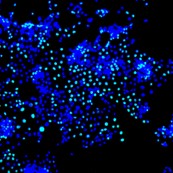

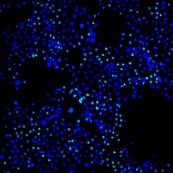

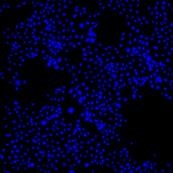

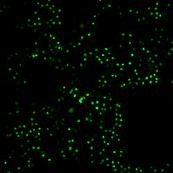

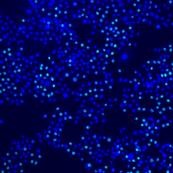

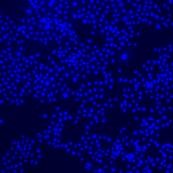

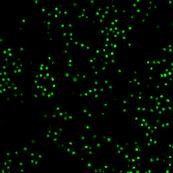

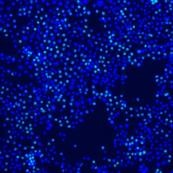

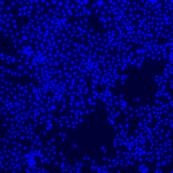

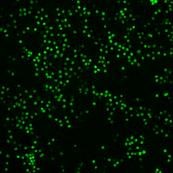


shSCR shBACH1shBACH1+IL-1β Vector BACH1 BACH1+siIL1B


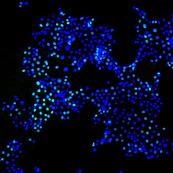

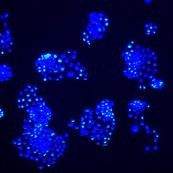

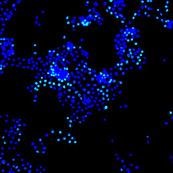

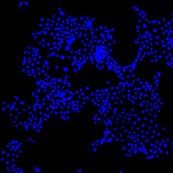

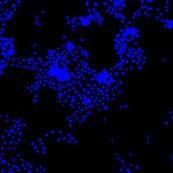

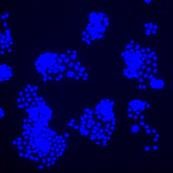

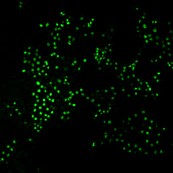

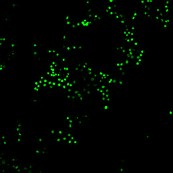

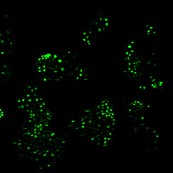


Hoechst

EdU

Merge


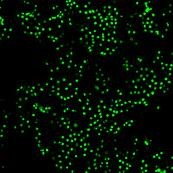

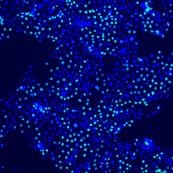

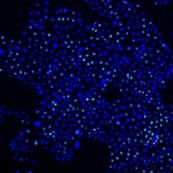

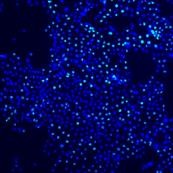

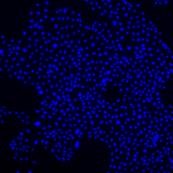

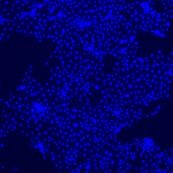

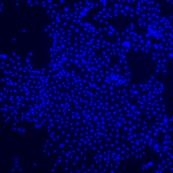

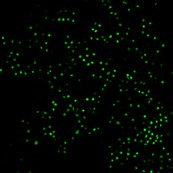

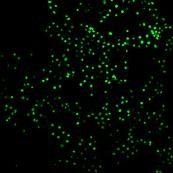


200



m
