# Supplementary figures and images for: A positive feedback loop between BACH1 and IL-1β promotes the progression of HPV-negative head and neck squamous cell carcinoma
Source: Cell Commun Signal. 2026 May 25;24:409. doi: 10.1186/s12964-026-02957-2 (PMC13377829; doi:10.1186/s12964-026-02957-2)

Supplementary Figure 2


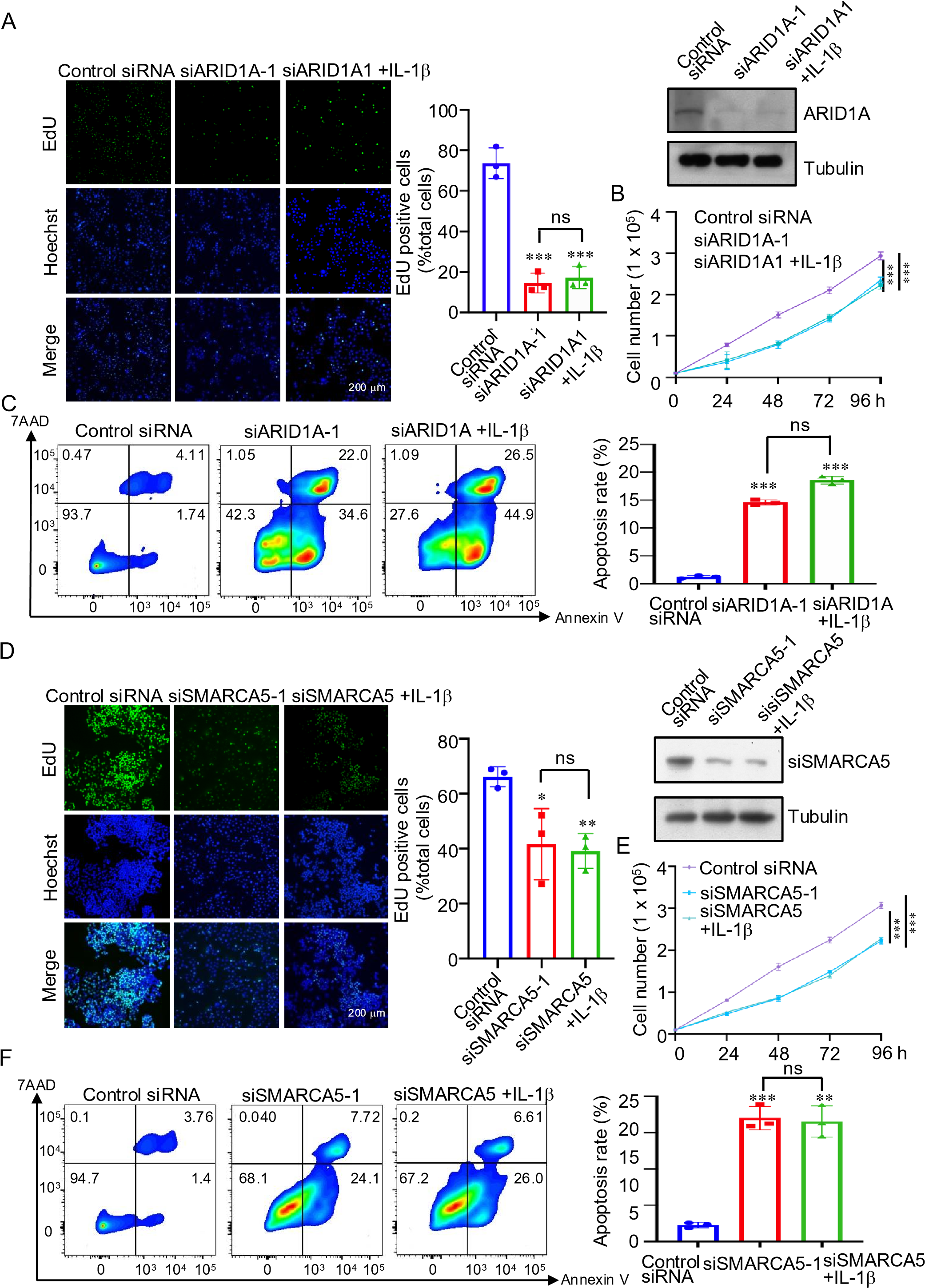

Supplement: Supplementary file 2 — Supplementary Material 2: Supplemental Figure 2, related to Figure 7. BACH1 orchestrates IL-1β production and maturation via the PBAF complex.EdU incorporation assay, growth curve, and apoptosis assay in FaDu cells following ARID1A knockdown treated with or without exogenous IL-1β. Scale bar: 200 μm. Error bars represent the mean ± SD from three independent experiments..EdU incorporation assay, growth curve, and apoptosis assay in FaDu cells following SMARCA5 knockdown treated with or without exogenous IL-1 β. Scale bar: 200 μm. Error bars represent the mean ± SD from three independent experiments. [file 12964_2026_2957_MOESM2_ESM.docx]

Supplementary Figure 3

A

PBS

Anakinra

PBS

Anakinra


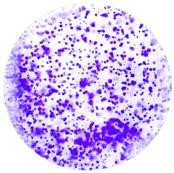

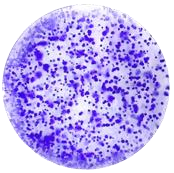

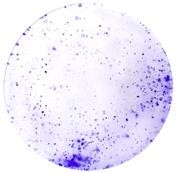

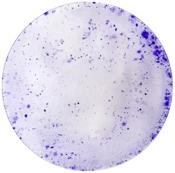


PBS

Anakinra


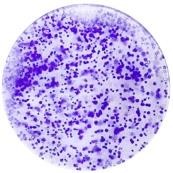

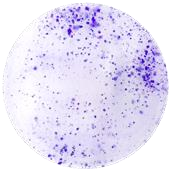

Supplement: Supplementary file 3 — Supplementary Material 3: Supplemental Figure 3, related to Figure 9. Interleukin-1 receptor antagonist Anakinra blocks HPV-negative HNSCC progression by targeting BACH1-IL-1β The colony formation assay performed in FaDu cells treated with PBS or Anakinra, shown as three independent replicates. [file 12964_2026_2957_MOESM3_ESM.docx]
