# Supplementary material for: A positive feedback loop between BACH1 and IL-1β promotes the progression of HPV-negative head and neck squamous cell carcinoma
Source: Cell Commun Signal. 2026 May 25;24:409. doi: 10.1186/s12964-026-02957-2 (PMC13377829; doi:10.1186/s12964-026-02957-2)
Supplement: Supplementary file 4 — Supplementary Material 4. [file 12964_2026_2957_MOESM4_ESM.docx]

**Supplementary Table 1 qCHIP primers**

| Gene | Primer (5′→3′) | |
| --- | --- | --- |
| BACH1 | Forward primer | AGTGTAAACTCCGCAGGTATCA |
|  | Reverse primer | TTTGGGGCATAAAGAAGGCAA |
| BRG1 | Forward primer | CAGATCCGTCACAGGCAAAAT |
|  | Reverse primer | TCTCGATCCGCTCGTTCTCTT |
| IL1B | Forward primer | TTCGACACATGGGATAACGAGG |
|  | Reverse primer | TTTTTGCTGTGAGTCCCGGAG |
| IL6 | Forward primer | CGGGTCCTGAAATGTTAT |
|  | Reverse primer | ATCGGTTTCTTTGCTTTG |
| MYC | Forward primer | GGCTCCTGGCAAAAGGTCA |
|  | Reverse primer | CTGCGTAGTTGTGCTGATG |
| PIM2 | Forward primer | TCCCGTGGAGTTGTCCATC |
|  | Reverse primer | GGCACCAGAACCAAAATCAATG |
| HSPA9 | Forward primer | GGAAGGTAAACAAGCAAAGGTGC |
|  | Reverse primer | CCAACAAGTCGCTCACCATCT |
| SIRT1 | Forward primer | CTGTTTCCTGTGGGATACCTGACI |
|  | Reverse primer | ATCGAACATGGCTTGAGGATCT |
| EGFR3 | Forward primer | TCCTGTTTTGTCTCCCCTTACG |
|  | Reverse primer | TCAGGATCTGGCAGAAGACGAT |
| MDM2 | Forward primer | GAATCATCGGACTCAGGTACATC |
|  | Reverse primer | TCTGTCTCACTAATTGCTCTCCT |
| USP13 | Forward primer | TGACGATTTAAATAGCGACGATTA |
|  | Reverse primer | GTCCTGCTTTCTGTATGGAGATTI |
| SMAD5 | Forward primer | CCAGCAGTAAAGCGATTGTTGG |
|  | Reverse primer | GGGGTAAGCCTTTTCTGTGAG |

**Supplementary Table 2 siRNA sequence**

| Name | Sequence (5'-3') | |
| --- | --- | --- |
| siBACH1-1 | Sense strand | CCAGCAAGAAUGCCCAAGAAATT |
|  | Antisense strand | UUUCUUGGGCAUUCUUGCUGGTT |
| siBACH1-2 | Sense strand | GUGCCAGUCAGACAUAUGATT |
|  | Antisense strand | UCAUAUGUCUGACUGGCACTT |
| siBRG1-1 | Sense strand | GGUGGACUACAGCGACUCATT |
|  | Antisense strand | UGAGUCGCUGUAGUCCACCTT |
| siBRG1-2 | Sense strand | GGCUUGAUGGAACCACGAATT |
|  | Antisense strand | UUCGUGGUUCCAUCAAGCCTT |
| siIL1B-1 | Sense strand | GGUGAUGUCUGGUCCAUAUTT |
|  | Antisense strand | AUAUGGACCAGACAUCACCTT |
| siIL1B-2 | Sense strand | GCGUGUUGAAAGAUGAUAATT |
|  | Antisense strand | UUAUCAUCUUUCAACACGCTT |

**Supplementary Table 3 qPCR primers**

| Gene | Primer (5′→3′) | |
| --- | --- | --- |
| IL1B | Forward primer | AGGAACCAGGAGGAGCAA |
|  | Reverse primer | TGGGATTACAGGCGTGAG |
| MYC | Forward primer | GCAAACTCAACGGGTAAT |
|  | Reverse primer | TCATCCTTGGTCCCTCAC |
| PIM2 | Forward primer | GGAAGAACCGAGCGTGTA |
|  | Reverse primer | CGCCCACCTGGCTTTGAT |
| HSPA9 | Forward primer | GCCGCAGTTAGTCTCCACC |
|  | Reverse primer | TTACAGTCTTCTTGCGTCAGTT |
| IL6 | Forward primer | CGGGTCCTGAAATGTTAT |
|  | Reverse primer | ATCGGTTTCTTTGCTTTG |
