# Supplementary material for: A positive feedback loop between BACH1 and IL-1β promotes the progression of HPV-negative head and neck squamous cell carcinoma
Source: Cell Commun Signal. 2026 May 25;24:409. doi: 10.1186/s12964-026-02957-2 (PMC13377829; doi:10.1186/s12964-026-02957-2)

Figure 2A

NC shBACH1

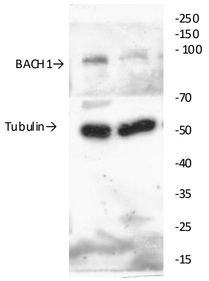

Figure 2A

VEC BACH1

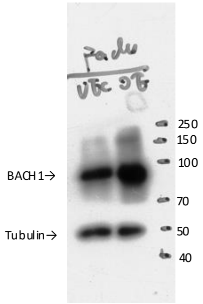

Figure 2H Up

NC +Hemin

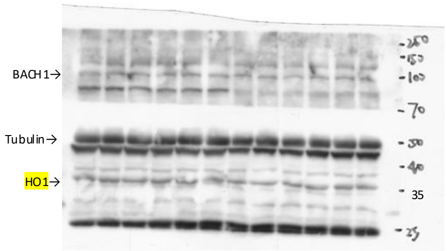

Figure 2E siBACH1

NC si-1 si-2

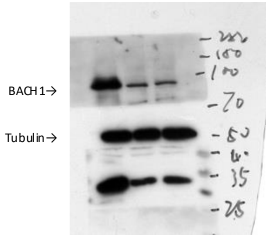

Figure 2H Bottom

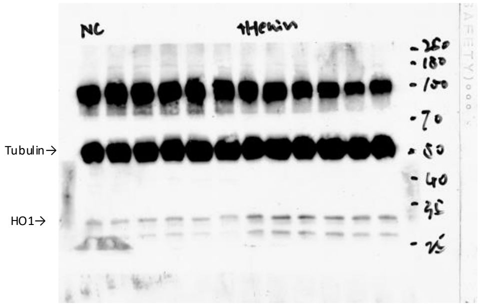

Figure 3A

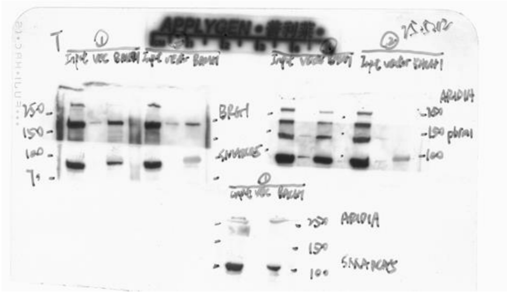

Figure 3B-FADU LEFT PBRM1

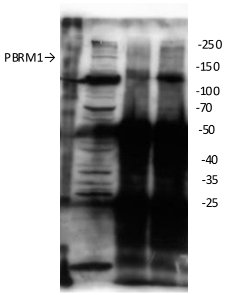

Figure 3B-FADU LEFT BRG1

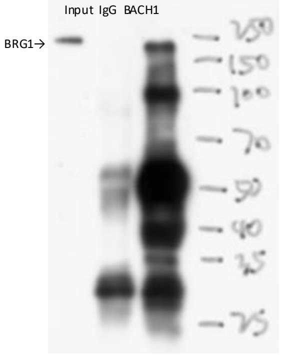

Figure 3B-FADU LEFT ARID1A

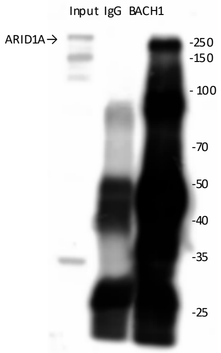

Figure 3B-FADU RIGHT ARID1A

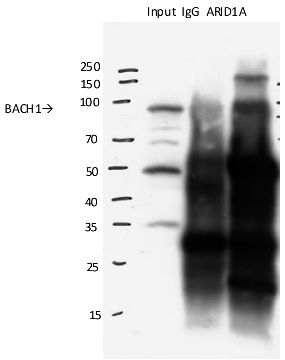

Figure 3B-CAL27 RIGHT PBRM1

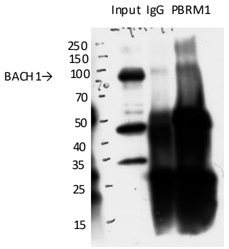

Figure 3B-293T LEFT BRG1

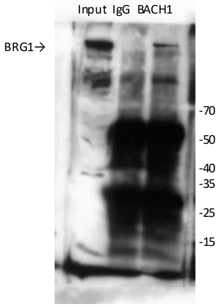

Figure 3B-293T LEFT PBRM1

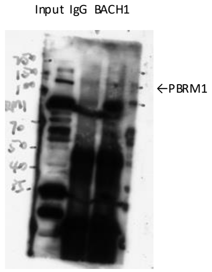

Figure 3B-293T LEFT ARID1A

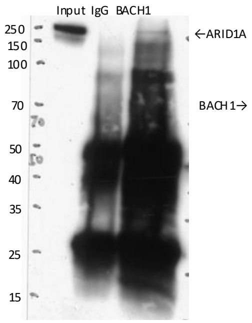

Figure 3B-293T Right ARID1A

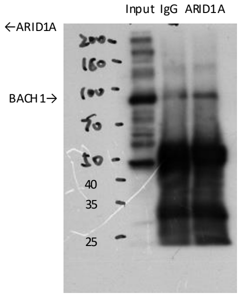

Figure 3B-293T Right PBRM1

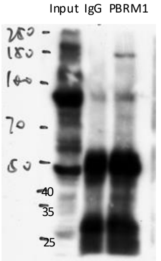

Figure 3B-293T Right BRG1

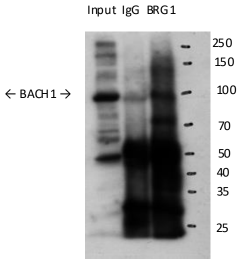

Figure 3B-FADU RIGHT BRG1

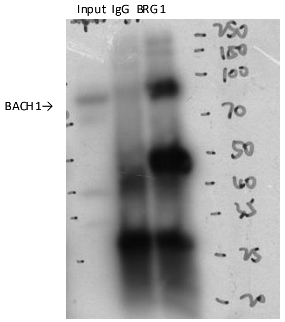

Figure 3B-FADU RIGHT PBRM1

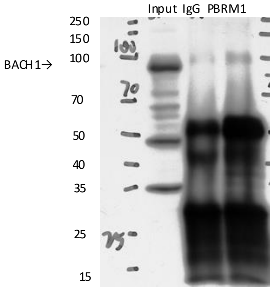

Figure 3B-CAL27 LEFT PBRM1

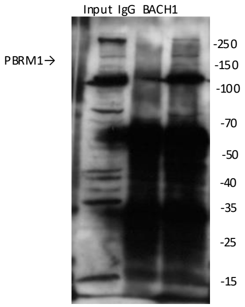

Figure 3B-CAL27 LEFT BRG1

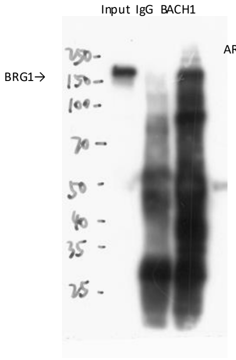

Figure 3B-CAL27 LEFT ARID1A

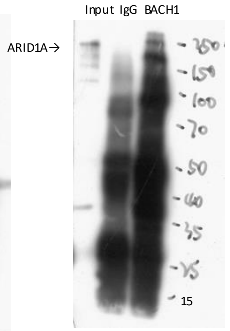

Figure 3B-CAL27 RIGHT BRG1

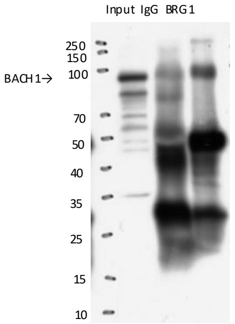

Figure 3B-CAL27 RIGHT ARID1A

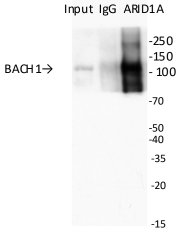

Figure 3C

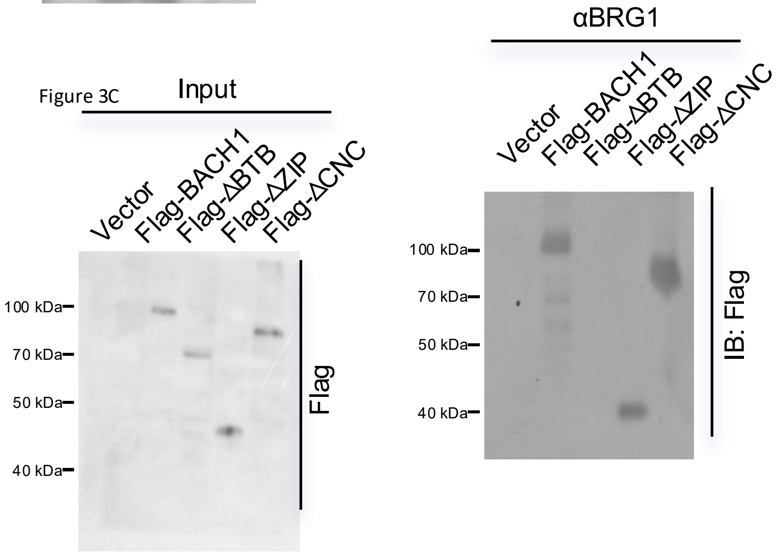

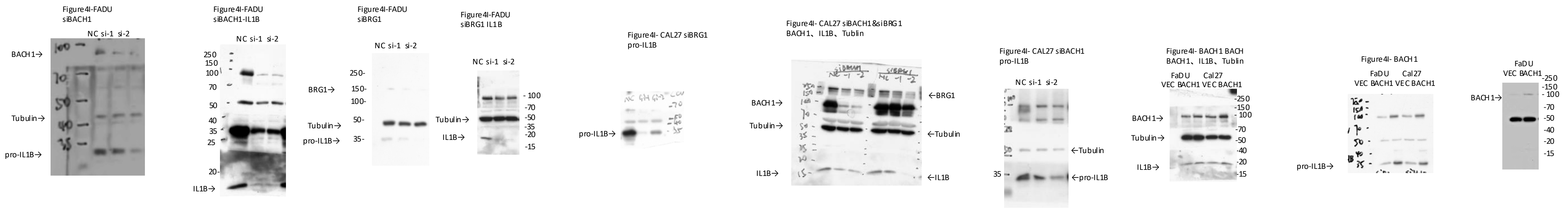

Figure4I-FADU siBACH1
NC si-1 si-2
BACH1→
Tubulin→
pro-IL1B→
Figure4I-FADU siBACH1-IL1B
NC si-1 si-2
250
150
100
70
50
40
35
25
20-
IL1B→
Figure4I-FADU siBRG1
NC si-1 si-2
250-
150-
100-
50-
35-
BRG1→
Tubulin→
pro-IL1B→
Figure4I-FADU siBRG1 IL1B
NC si-1 si-2
Tubulin→
IL1B→
- 100
-70
-50
-35
-20
-15
Figure4I- CAL27 siBRG1 pro-IL1B
pro-IL1B→
Figure4I- CAL27 siBACH1&siBRG1 BACH1、IL1B、Tublin
BACH1→
Tubulin→
IL1B→
←BRG1
←Tubulin
←IL1B
Figure4I- CAL27 siBACH1 pro-IL1B
NC si-1 si-2
35
←Tubulin
←pro-IL1B
Figure4I- BACH1 BACH BACH1、IL1B、Tublin
FaDU VEC BACH1
Cal27 VEC BACH1
BACH1→
Tubulin→
IL1B→
-250
-150
- 100
-70
-50
-40
-20
-15
Figure4I- BACH1
FaDU VEC BACH1
Cal27 VEC BACH1
pro-IL1B→
FaDU VEC BACH1
BACH1→
-250
-150
- 100
-70
-50
-40
-20
-15

Figure5A siIL1B

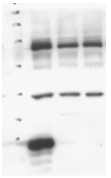

Figure6C

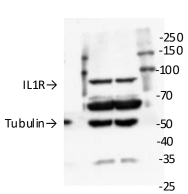

Figure6D

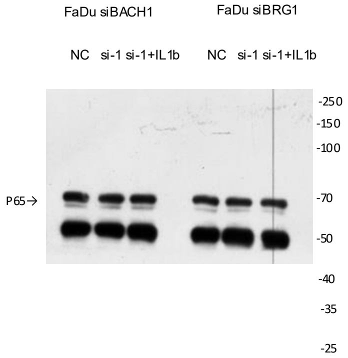

Figure6D

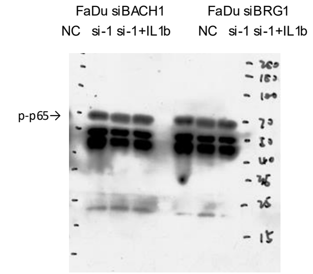

Figure6D

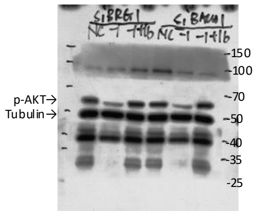

Figure6D

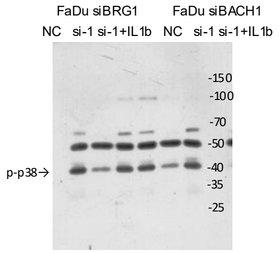

Figure6D

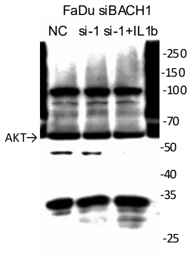

Figure6D

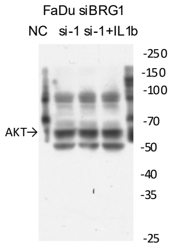

Figure6D

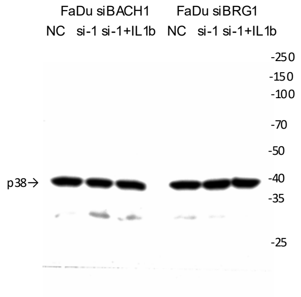

Figure6F

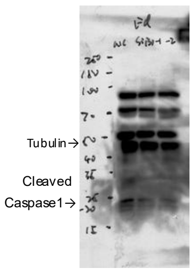

Figure6H-shBACH1

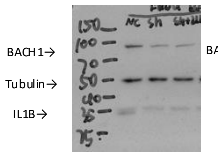

Figure6H-BACH1

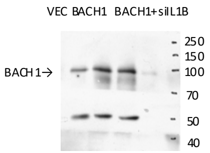

Figure6H-BACH1

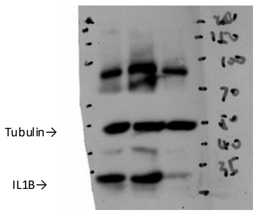

Figure 7C

CAL27 siPBRM1
NCsi-1 si-2

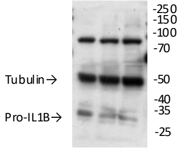

Figure 7C
CAL27 siPBRM1
NCsi-1 si-2

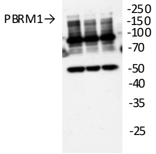

Figure 7C

CAL27 siARID1A
NCsi-1 si-2

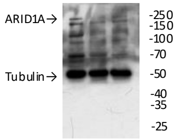

Figure 7C

CAL27 siARID1A
NCsi-1 si-2

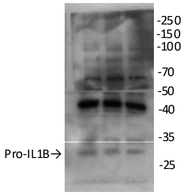

Figure 7C

Fadu siPBRM1
NCsi-1 si-2

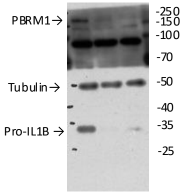

Figure 7C

Fadu si ARID1A
NCsi-1 si-2

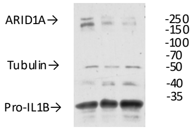

Figure 7F

Fadu siPBRM1
NCsi-1 si-2

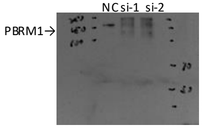

Fadu siPBRM1
NCsi-1 si-2

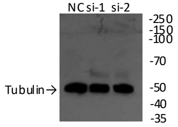

Figure8D-siBACH1

NC si-1 si-2

SIRT1→

Tubulin →

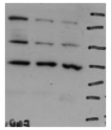

150
100
70
50

Figure8D-siBACH1

NC si-1 si-2

BACH1→

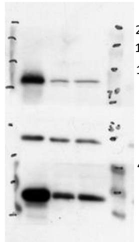

250
150
100
40
35

Figure8D-siBACH1

USP13→

MDM2→

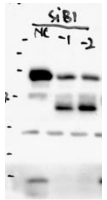

Figure8D-siIL1B

NC si-1 si-2

Pro-IL1B→

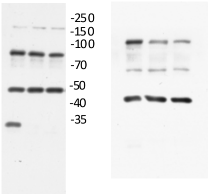

-250
-150
-100
-70
-50
-40
-35

NC si-1 si-2

←SIRT1

Figure8D-siIL1B

NC si-1 si-2 NC si-1 si-2

MDM2→

Tubulin→

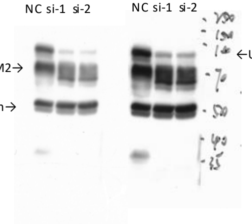

←USP13

Figure8E-siIL1B

NC si-1 si-2

BACH1→

Tubulin→

Pro-IL1B→

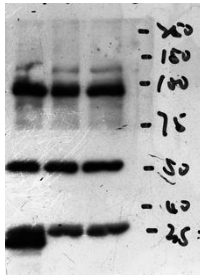

Figure9E
PBS Ana

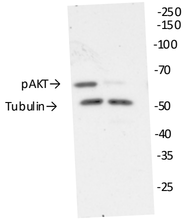

Figure9E
PBS Ana

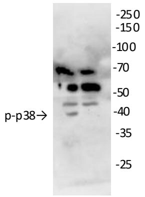

Figure9E
PBS Ana

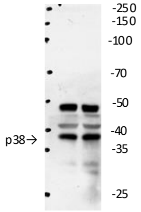

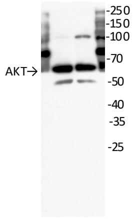

Supplementary Figure 2

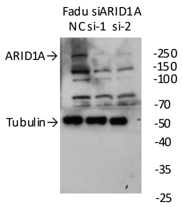

Supplementary Figure 2

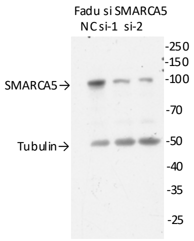

Supplement: Supplementary file 10 — Supplementary Material 10. [file 12964_2026_2957_MOESM10_ESM.pdf]
